# Determination of Methyl Group Positions in Long-Chain Aliphatic Methyl Ethers and Alcohols by Gas Chromatography/Orbitrap Mass Spectrometry

**DOI:** 10.1021/acs.analchem.5c03083

**Published:** 2025-07-29

**Authors:** Tatsuya Kiuchi, Moritz Gerbaulet, Anton Möllerke, Tim Harig, Axel Dinter, Till Beuerle, Stefan Schulz

**Affiliations:** a Institute of Organic Chemistry, Technische Universität Braunschweig, Hagenring 30, Braunschweig 38106, Germany; b Independent Researcher, Nelkenweg 14, Friedrichsdorf 61381, Germany

## Abstract

Methylated long-chain aliphatic compounds such as terminal methyl ethers are a common compound type found on the epicuticular layer of arthropods, e.g., spiders. Because complex mixtures are encountered in small amounts when analyzing these mixtures, GC/MS is the method of choice for characterizing the individual constituents. However, the methyl branch location cannot be deduced from the original spectra due to the easy loss of methanol, resulting in nonspecific spectra, and a complex derivatization scheme has been employed to address this issue. We noted that although mass spectra obtained by EI-quadrupol and EI-Orbitrap ionization are superficially quite similar, a +2.0 V C-trap offset of the latter leads to reduced fragmentation. The high-resolution Orbitrap spectra contain enough information to allow for methyl group localization in the chain. However, the spectra of the methyl ethers contain many ions, making individual analysis quite time-consuming. Therefore, scripts using Excel and R were developed with the help of ChatGPT 4.0, resulting in ion series spectra (ISS) that contained only ions of a specific ion series. The analysis of 11 synthetic methyl ethers showed that especially the ion series C_*n*_H_2*n*+1_O (ISS45) and C_*n*_H_2*n*–2_ (ISS40) are of high diagnostic value, together with some methoxy group-induced fragmentation. The approach was successfully tested with lipids from the spider , which had been previously analyzed by derivatization, and with web extracts of *Erigone atra*, revealing 1-methoxy-2,16-dimethylhenicosane as a male-specific component—the first spider methyl ether in a volatility range that would allow detection via the gas phase. This approach can also be applied to structurally related primary alcohols, although the diagnostic ions are of lower intensity.

## Graphical abstract

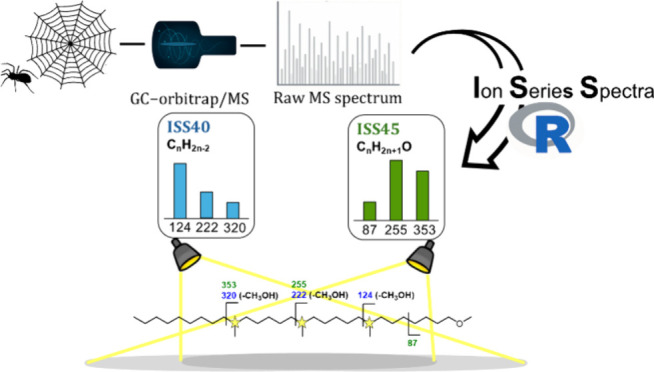

## Introduction

For several decades, electron ionization (EI) gas chromatography–mass spectrometry (GC-MS) mass spectra with their highly reproducible fragmentation enable powerful structure identification within databases, especially when combined with gas chromatographic retention index data.^1^ The standard database for this purpose is the Mass Spectral Library of the National Institute of Standards and Technology (NIST), which contains over 394,000 EI spectra and 491,000 retention index values.^2^ Traditional high-resolution time-of-flight (TOF) or sector field mass analyzers generate MS data that are analogous to those produced by standard quadrupole GC-MS instruments. In such instances, unit-mass resolution databases can be utilized without constraints, yielding results that demonstrate a high degree of agreement, often exceeding a match of 900 (as determined by NIST database searches; a match of 1000 signifies perfect identity). However, the identification does not benefit from the exploration of high-resolution, accurate mass GC-MS data in such unit-mass resolution databases. In the newer GC-Orbitrap instruments, which use a damping gas and a C-trap for ion storage prior to injection into the Orbitrap analyzer, unpredictable distortion of the EI spectra occurs, as has been frequently observed and reported.^3–5^ Based on our experience, this reduces the general match of GC-Orbitrap spectra against the NIST database to approximately 800.

This result was recently confirmed by a larger study comparing 480 EI-Orbitrap spectra vs NIST, and the observed match was, on average, 786, ranging from 270 to 937.^6^ The quality of the match is primarily influenced by the absence of lower *m*/*z* ions, variations in ion abundance (both positive and negative impact on fragment ions within a single spectrum), the presence or absence of ions, and the substantial reduction of the molecular ion in Orbitrap MS.^6^ This is particularly problematic for MS of alkanes, which are susceptible to suppression of the molecular ion. This phenomenon is counterproductive in cases in which the molecular formula cannot be derived from the molecular ion, particularly in the investigation of unknown compounds.

In such instances, the manufacturer’s recommendation is to apply a positive potential of +2.0 V as a C-trap offset in EI­(+)-MS. This is intended to pack the ions more tightly within the C-trap, thereby reducing the available volume for damping gas collisions. This, in turn, results in better preservation of the fragile molecular ions.

This method is a convenient approach for obtaining the desired molecular mass information for classes of compounds that show no abundant molecular ion, such as alkanes, alcohols, or methyl ethers. However, regaining the molecular ion peak will not invert the general distortion of the Orbitrap mass spectra. It might positively affect the sorting of the database hits but not necessarily restore a better match in the database search.

Long-chain aliphatic hydrocarbons and their derivatives, such as alcohols, their esters, or aldehydes, constitute the outermost wax layer of insects, but also of other arthropods such as arachnids.^7,8^ These compounds, usually occurring in complex mixtures, are composed mostly of between 20 and 45 carbons and are often methyl-branched at certain positions along the chain. While methyl group positions in alkanes can be relatively straightforwardly determined by analyzing EI-mass spectra obtained from GC/MS analyses of cuticular hydrocarbon extracts,^9^ the situation changes in oxidized derivatives. Alcohols and aldehydes easily lose water under EI conditions, and the resulting mass spectra do not allow the determination of the methyl group positions along the chain. The same is true for respective methyl ethers that lose methanol. For all these compounds except alkanes, methyl group positions are usually determined by derivatization, followed again by GC/MS. Thus, alcohols can be transformed into nicotinates,^10^ while methyl ethers need to be first transformed into methyl esters and iodides, which can then be converted into nitriles.^11^ The resulting mass spectra indicate methyl group positions by missing ions in a specific, often N-stabilized ion series, leading to a gap of 28 u instead of the normal 14 u.

Methyl ethers are a characteristic class of wax components on the cuticle of many spiders,^12–16^ where they can function in species recognition.^13,14^ The lipid layer is also important for using spider silk in medical applications, where it is under consideration as guidance material for nerve regeneration.^16^ The thorough investigation of the composition of such lipid mixtures is usually hampered by the limited amounts of material available, making derivatization procedures difficult. Furthermore, derivatization can lower the sensitivity and introduce impurities and discrimination problems. Not all derivatization steps may be similarly effective on all constituents of a sample, potentially leading to altered relative concentrations of the sample constituents. Therefore, a direct determination of the methyl group position of the original mass spectra would be beneficial.

In our study, we investigated mass spectra of terminal long-chain alkyl methyl ethers by GC-orbitrap/MS, applying the C-trap offset. We reasoned that the distorted spectra obtained might reveal ions indicative of methyl group positions, probably absent or of low intensity in GC/EI-MS analyses of methyl ethers. Fortunately, a range of synthetic methyl ethers were available from our in-house compound library, allowing this investigation.

## Experimental Section

### Compounds

The analyzed methyl ethers were available from our in-house compound collection, which originated from our research on spider lipids and the cuticular chemistry of arthropods.^12,13,15–19^

### Gas Chromatography

A Thermo Scientific Trace 1310 gas chromatograph (Thermo Scientific, Bremen, Germany) was equipped with a 30 m analytical column (Phenomenex ZB5-MS, 30 m × 0.25 mm ID, *t*_f_ = 0.25 μm). A split/splitless injection port at 270 °C was used for sample introduction in splitless mode. The temperature program was: 100 °C (3 min)–5 °C/min-320 °C (3 min). The helium carrier gas was set to a flow rate of 1.0 mL/min (constant flow mode). Linear retention indices^20^ were measured using a series of *n*-alkanes and calculated according to our previously published procedure.^13,15^

### Mass Spectrometry

An Exactive GC-Orbitrap mass spectrometer (ThermoScientific, Bremen, Germany) was used. The resolution was set to 60,000 (fwhm; instrument setting at 200 u). The mass range was 40–500 u, and 2 micro scans were averaged per data scan. The automated gain control (AGC target) was set to 1 × 1 × 10^6^, and the maximum inject time was set to “auto”. The auxiliary temperatures were set to 290 °C for transfer lines 1 and 2, and the electron ionization source temperature was set to 220 °C. EI was performed at 70 eV energy in positive mode. Helium (carrier gas) and nitrogen (supply for the C-trap) were equipped with gas purification cartridges to trap moisture and organic impurities of the gases (Thermo Scientific, Bremen, Germany). The C-trap energy offset for these measurements was 2.0 V. The column bleed ion at 207.03235 u was used as the lock mass for internal mass calibration of the data.

### Spider Rearing and Web Collection

 (Blackwall) females were collected by hand in fields near Friedrichsdorf (Hessen, Germany). The spiders were kept in glass tubes (100 mm × 23 mm) filled with a small layer of moist plaster of Paris at 20 °C and a L16:D8 regime and fed twice a week with live fruit flies ( L.). The egg cocoons produced by females were separated in glass tubes filled with a small layer of moist Paris plaster until the spiderlings hatched. 1 to 2 days old were placed individually in glass tubes filled with a small layer of moist compost soil containing hundreds of Collembola, specifically (Gmelin), as food items. Additionally, the spiderlings were fed live fruit flies twice a week until they reached the adult stage.^21,22^ About 1 week-old males and females were transferred individually into glass tubes filled with a small layer of moist plaster of Paris. The webs produced by the spiders were collected at 1–2 day intervals using a metal wire and stored in small glass tubes at −18 °C. For silk extracts, five webs were combined.

### Lipid Extracts

Extracts of were obtained as described previously.^13^ The silk samples of were placed in a 1 mL vial with an insert, and 20 μL of dichloromethane (Suprasolv, Merck) was added. The samples were stored at −80 °C until analysis. For GC/MS analysis, 1 μL was injected into the gas chromatograph.

### General Procedure for Methyl Group Position Determination

This procedure was applied in the final section of silk extracts. It makes it more convenient to locate methyl groups.

1. According to HR-MS and RI, the carbon number of the longest chain and the number of methyl groups are calculated.
2. Check whether *m*/*z* 87 and 101 are present to identify a methyl group between C-2 and C-4. *m*/*z* 101 indicates a methyl group. Check the presence of ion **h**. If present, a C-4-methyl is present.
3. Check ISS45, ions **a**, to identify the location of the methyl group, for example, in Figure 3, *m*/*z* 213.
4. Check ISS40, ions **d** and **h**, to support (3), for example, as shown in Figure 3, where *m*/*z* 264 and 180 are observed.
5. Unless the number of methylation based on (3) ISS45 did not match with (1), pick intense peaks in ISS40 not used at (4) and analyze them.

## Results and Discussion

During the analysis of alkyl methyl ethers from spider lipids, we observed that quadrupole unit-resolved EI-mass spectra differed from those obtained with an Orbitrap ion source, for example, for 1-methoxyhexacosane (Figure 1). While superficially the spectra look similar, differences can be observed, especially in the intensity of the ions of higher masses above *m*/*z* 150, which are more intense in the Orbitrap spectrum. Mass spectra of methyl ethers show an easy loss of methanol, leading to ion *m*/*z* 364 as the highest ion with a larger intensity. However, because of the obviously lower fragmentation in the Orbitrap spectrum (Figure[Fig fig1]B), we reasoned that ions still containing O might be better visible in the Orbitrap spectrum. In addition, the Orbitrap high-resolution data would allow for definite proof of their molecular formula. However, the analysis of the whole spectrum is time-consuming since a long list of ions or parts of the spectrum must be checked manually. Therefore, we introduce here ion series spectra (ISS), showing only ions of a specific series, to facilitate the discussion. With the help of ChatGPT-generated Excel macros and R-coding (see Supporting Information), we extracted specific ion series from the spectral data and displayed them as ISS (Figure 1C–F). An ISS45 contains all ions of the series C_*n*_H_2*n*+1_O, while ISS40 shows the ion series C_*n*_H_2*n*–2_.

**Figure 1:**
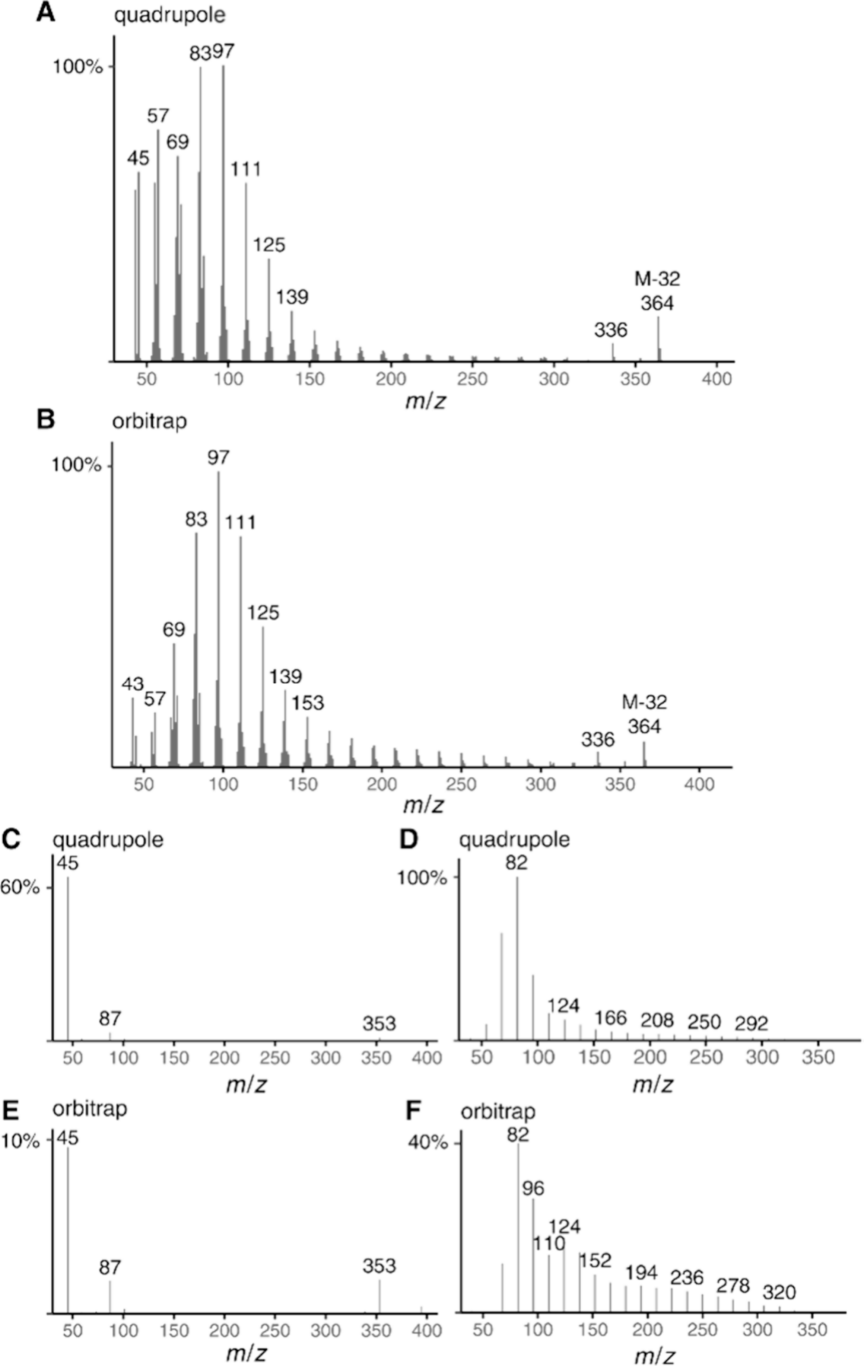
Mass spectra of 1-methoxyhexacosane. (A) EI-quadrupole; (B) EI-Orbitrap; C: ion series spectrum ISS45 (C_*n*_H_2*n*+1_O) quadrupole; (D) ion series spectrum ISS 40 (C_*n*_H_2*n*–2_) quadrupole; (E) ion series spectrum ISS45 (C_*n*_H_2*n*+1_O) Orbitrap; (F) ion series spectrum ISS40 (C_*n*_H_2*n*–2_) Orbitrap.

Few O-containing ions occur in the mass spectrum of linear methyl ethers (Figure 1C,E). While *m*/*z* 45 is formed by simple α-fragmentation, indicating the methyl ether functionality, ions *m*/*z* 87 and 353 are formed by rearrangement. Ionization of O can lead to δ-fragmentation, resulting in the formation of ion *m*/*z* 87, which is stabilized by ring formation (Scheme 1). Correspondingly, ion *m*/*z* 353 (M-43) can be explained by the loss of a propyl group. This propyl group likely originates from ions C-2 to C-4. A similar loss has been observed in the spectra of fatty acid esters.^23^ Supposedly, alkyl chain transfer, instead of H-transfer that leads to the ion M-32, induces a rearrangement. A subsequent H-transfer from the chain saturates the primary radical. Finally, α-cleavage leads to a ring-stabilized cation and release of a propyl radical (Scheme 1).

**Scheme 1:**
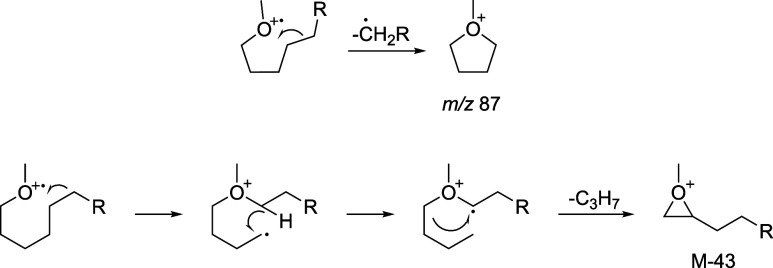
Proposed Fragmentation Leading to Ions *m*/*z* 87 and [M-43]^+^ ^a^

a The ring size can differ in the lower reaction pathway depending on the location of the H-abstraction. *n*= 0, 1, 2, ....

Given these prerequisites, the spectra of several synthetic long-chain methyl-branched alkyl methyl ethers available in our compound collection, due to our work on spider lipids, were then reanalyzed.^11–13,15^ The mass spectra of 1-methoxy-12-methylnonacosane are shown in Figure 2. The peak groups around *m*/*z* 180 and 264 are of higher abundance in the Orbitrap spectrum. The different ISS for both ionization types were compared in detail. The ISS of C_n_H_2n+1_O (ISS45, Figure 3B) in the Orbitrap spectrum showed the expected ions *m*/*z* 45, 87, and 409 (M-43), but additionally *m*/*z* 213, corresponding to cleavage next to the methyl group at C-12 (ion **a**, Figure 3A). Although the quadrupole ISS45 shows the same ions (Figure 3C), additional ions obscure the results, making a proper determination of the methyl group position less obvious. Further support stems from characteristic ions originating from alkyl fragmentation.^12^ A first loss of methanol yields an alkene that cleaves preferentially next to the methyl branch, leading to peak clusters due to additional H loss. The different ISS of these clusters have an individual appearance. While ISS42 and ISS41 do not clearly indicate the methyl group location, this is different for ISS40 (Figure 3D–I). The ions **d** *m*/*z* 180 and **h** *m*/*z* 264 clearly indicate the C-12 methyl group in the Orbitrap spectrum, while *m*/*z* 264 is not so clear in the original quadrupole spectrum, which shows *m*/*z* 266 to be more intense.

**Figure 2:**
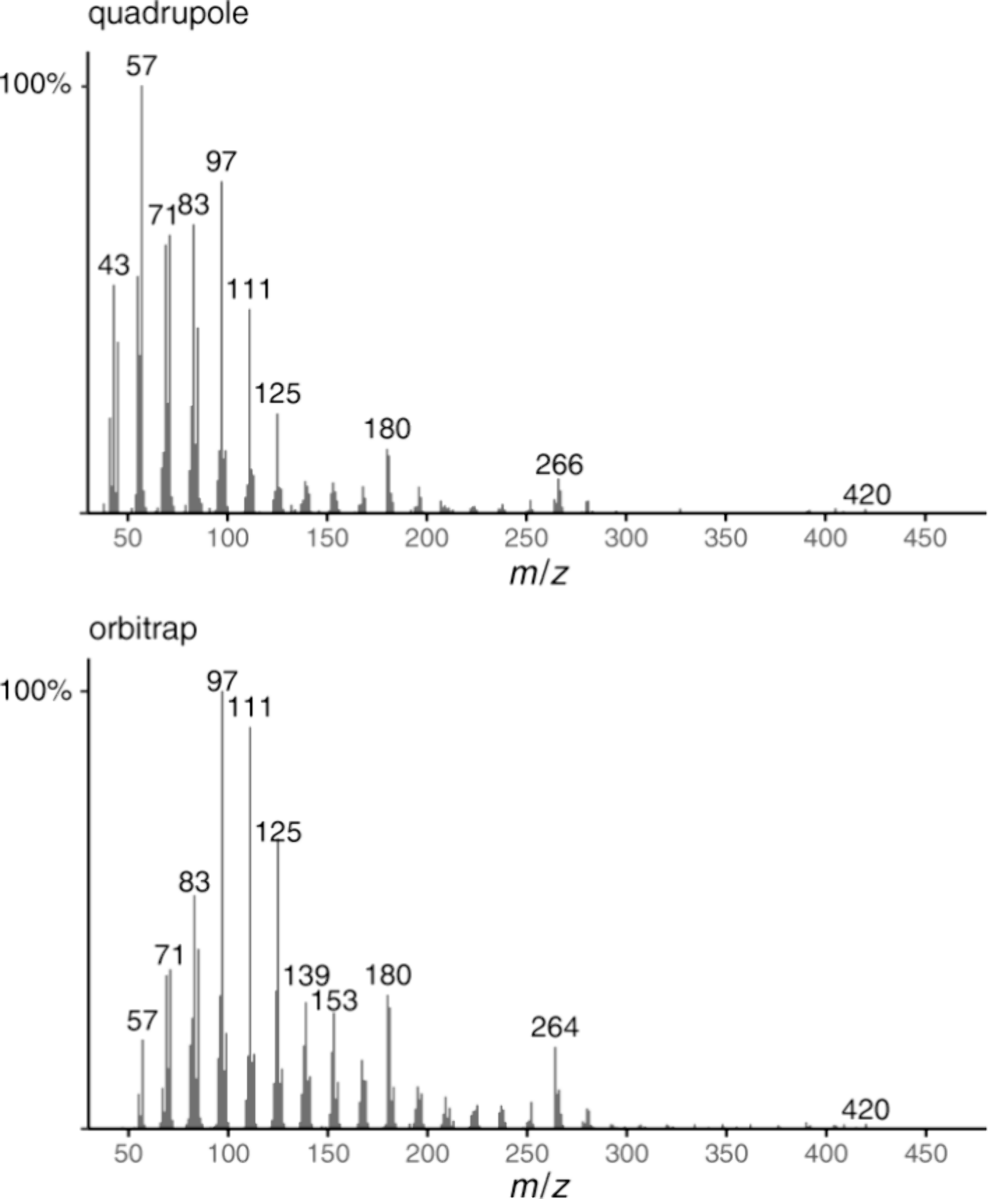
Mass spectra of 1-methoxy-12-methylnonacosane (**1**) obtained by EI-quadrupole (upper) or EI-Orbitrap ionization (lower).

**Figure 3:**
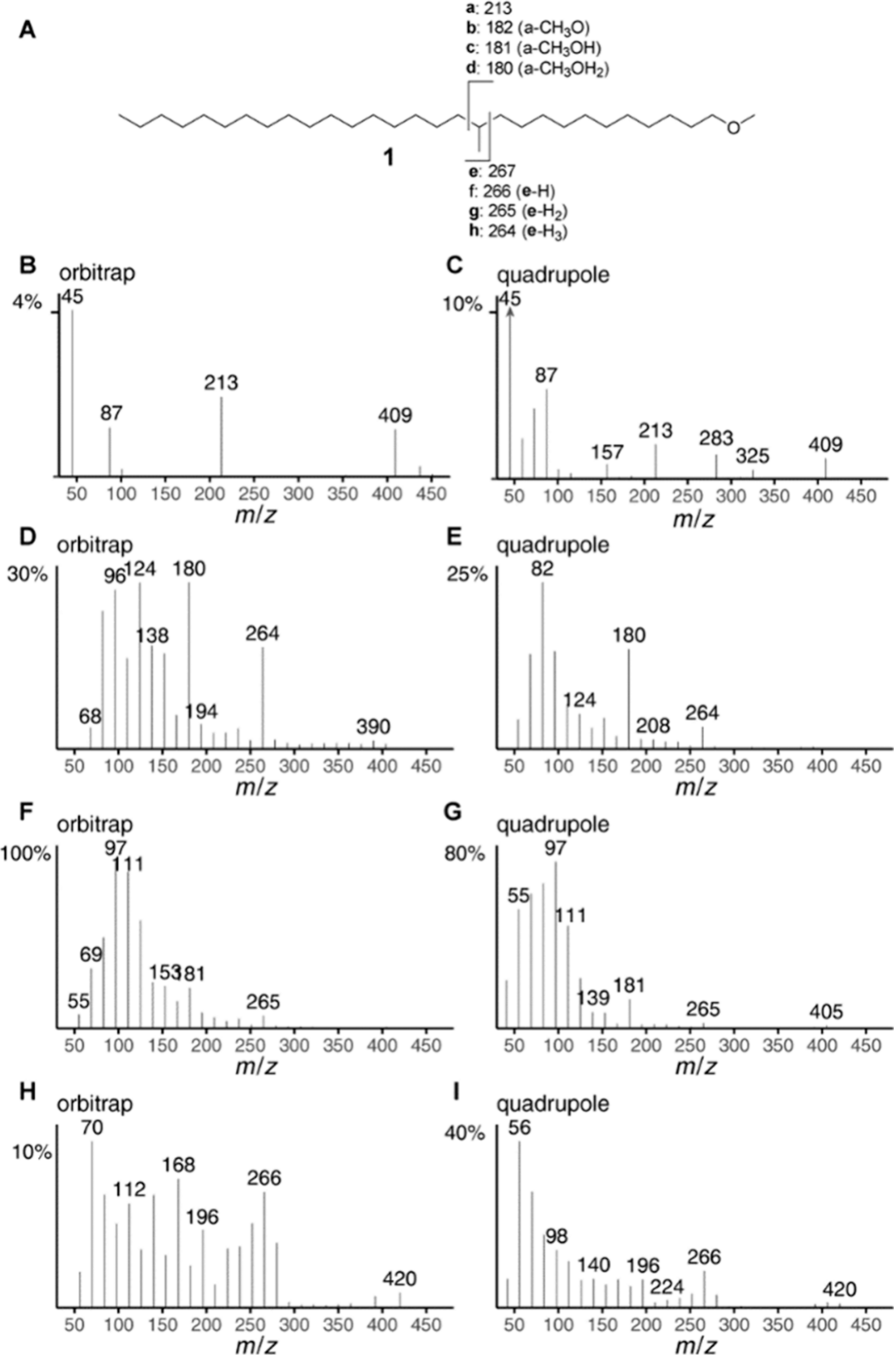
Fragmentation (A) and ion series spectra of 1-methoxy-12-methylnonacosane (**1**). (B, C) ISS45 of C_*n*_H_2*n*+1_O; (D, E) ISS40 of C_*n*_H_2*n*–2_; (F, G) ISS41 of C_*n*_H_2*n*‑1_; (H, I) ISS42 of C_*n*_H_2*n*_.

In summary, the Orbitrap spectrum was easier to interpret and provided a clearer indication of the methyl group’s location compared to the quadrupole spectrum. Of key importance were ISS45 and ISS40. We then evaluated this approach with other synthetic methyl-branched ethers, focusing on these ion series.

The original spectra as well as ISS45 and ISS40 spectra of 1-methoxy-24-methylheptacosane (**2**) and 1-methoxy-2,24-dimethylheptacosane (**3**) are shown in Figure 4. Ions **a** at *m*/*z* 381 and 395 indicate the methyl group at C-24, supported by ions **d** at *m*/*z* 348 and 362. The additional methyl group in **3** must be located between C-2 and C-4 because ion *m*/*z* 87 shifts to 101, and instead of M-43, M-57 occurs at *m*/*z* 381. An exact location of the methyl group can not be made because ions **h** were not clearly observed.

**Figure 4:**
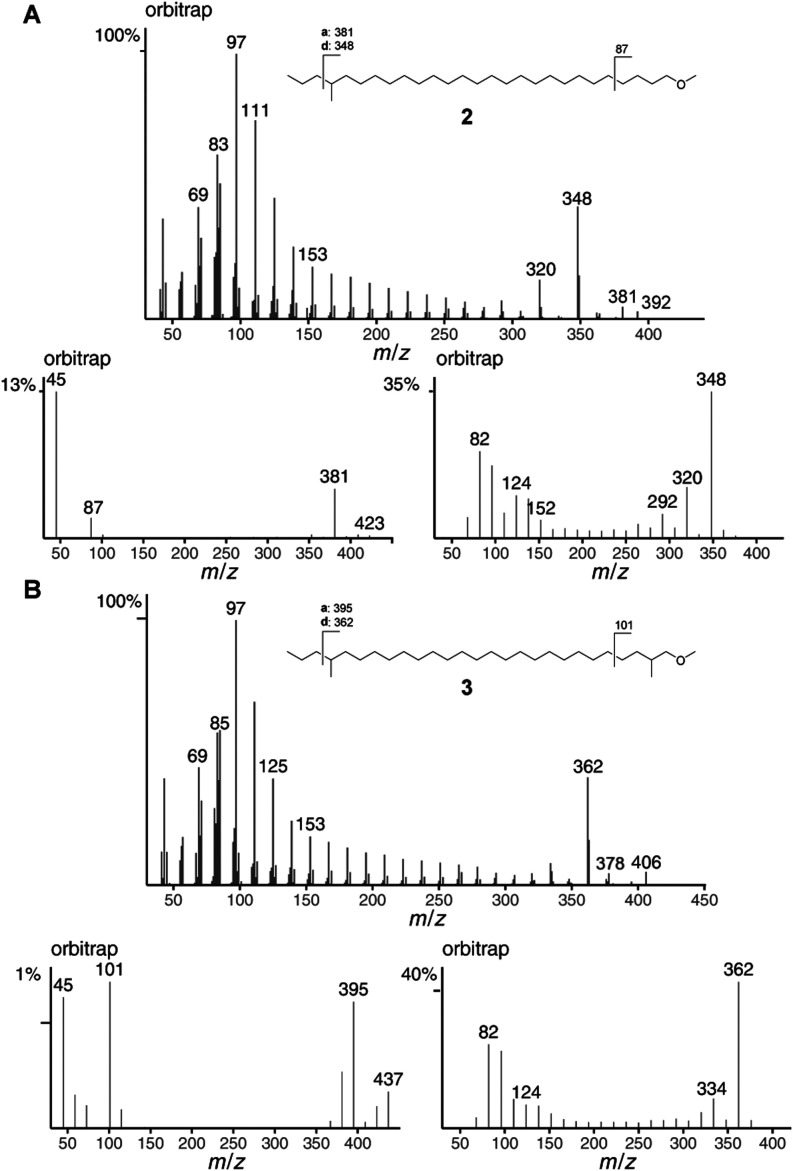
Orbitrap mass spectra, ISS45, ISS40, and fragmentation of (A) 1-methoxy-24-methylheptacosane (**2**) and (B) 1-methoxy-2,24-dimethylheptacosane (**3**).

In addition, several dimethylated and trimethylated ethers were analyzed accordingly. The ISS40 and ISS45 of 1-methoxy-12,20-dimethylnonacosane, 1-methoxy-14,20-dimethylnonacosane, 1-methoxy-14,19-dimethyloctacosane, and 1-methoxy-4,16-dimethylhenicosane are shown in Figure 5. ISS45 of 1-methoxy-12,20-dimethylnonacosane shows the expected ions **a** at *m*/*z* 213 and 339, while ISS40 confirms the methyl group position with ions **d** at *m*/*z* 180 and 306 and **h** at *m*/*z* 152 and 278. Similarly, ions **a** (*m*/*z* 241/339 and *m*/*z* 241/325), **d** (*m*/*z* 208/306 and *m*/*z* 208/292) and **h** (*m*/*z* 152/250 and *m*/*z* 152/236) indicate methyl group positions in 1-methoxy-14,20-dimethylnonacosane and 1-methoxy-14,19-dimethyloctacosane, although *m*/*z* 236 is not particularly high in ISS 40 of the latter (Figure 5F). The spectrum of 1-methoxy-4,16-dimethylhenicosane indicates the methyl group at C-16 through ions *m*/*z* 283 (**a**), and 250 (**d**), although *m*/*z* 222 becomes the most intense peak in this ion series. This ion, **d′**, is likely formed by a cleavage on the opposite side of the C-16-methyl group. Such ions, **d**′**** and similarly **h**′****, are also observed in the other ISS40 discussed but usually with lower intensity. Although *m*/*z* 101 does not clarify the position of the methyl group between C-2 and C-4, as discussed above, ion *m*/*z* 278 (**h**) confirms the C-4 position. The lack (C-2) or presence (C-4) of ion **h** thus makes differentiation between 2-methyl and 4-methyl ethers possible.

**Figure 5:**
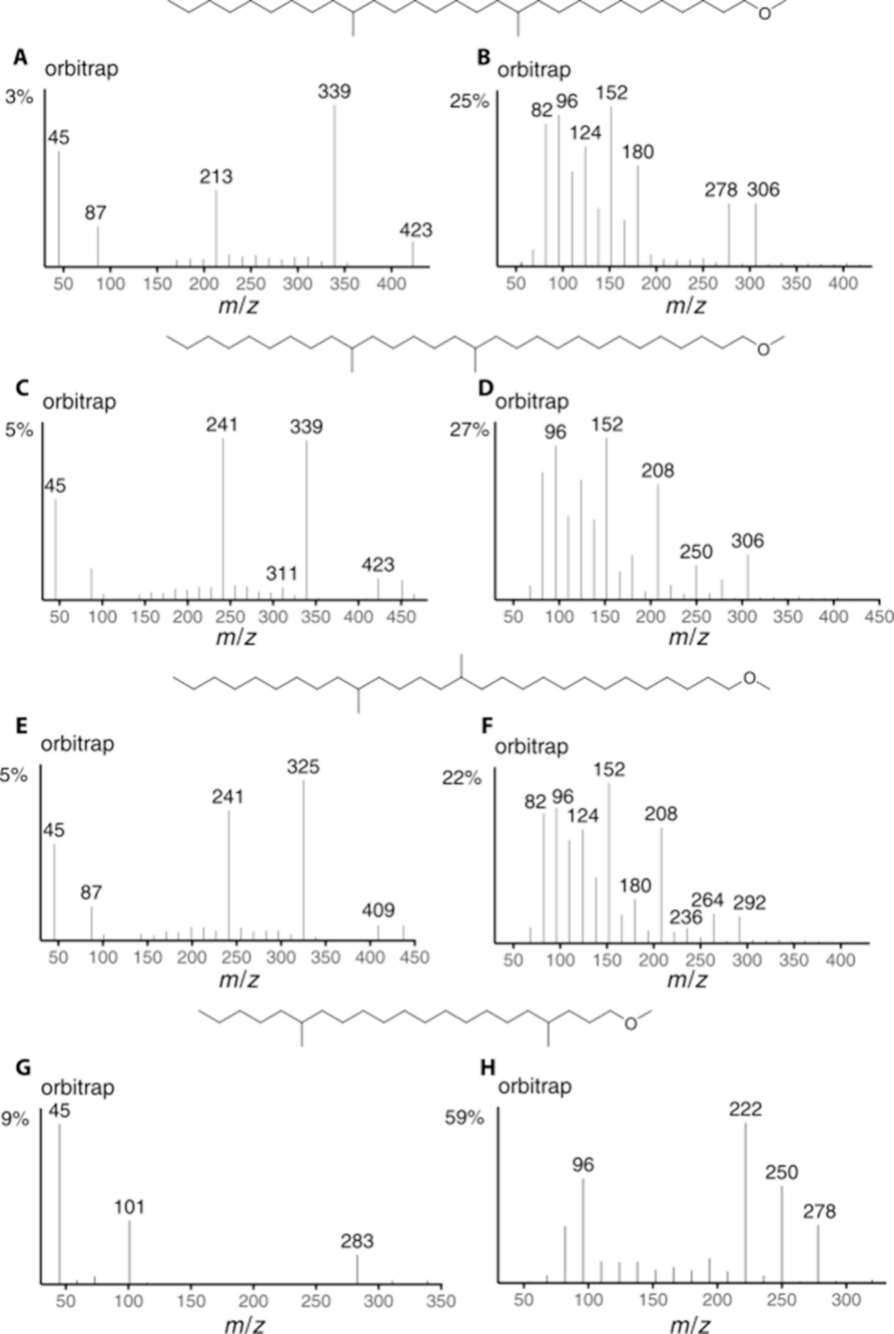
Orbitrap ISS45 (left) and ISS40 (right) of 1-methoxy-12,20-dimethylnonacosane (A, B), 1-methoxy-14,20-dimethylnonacosane (C, D), 1-methoxy-14,19-dimethyloctacosane (E, F), and 1-methoxy-4,16-dimethylhenicosane (G, H).

The applicability of this approach was extended to even higher methylated ethers. 1-Methoxy-17,21,25-trimethylhexacosane (Figure 6A,B) showed indicative ions for C-17-Me at *m*/*z* 283 (**a**) and 250 (**d**), although ion **h** (*m*/*z* 180) missed significance. Methyl-C-21 is supported by the ions at *m*/*z* 353, 320, and 110. As found in many mass spectra of *iso*-compounds, the respective methyl group C-25 cannot be located. Although *m*/*z* 423 was present, it may have originated from any loss of methyl, and ion **h** is missing. The spectrum of 1-methoxy-2,10,24-trimethylheptacosane (Figure 6C,D) was very similar to that of **3**, with *m*/*z* 409 and 376 (**d**) indicating the C-24 methyl group. The abundance of *m*/*z* 199 is relatively low, but is indicative when seen together with *m*/*z* 166 (**d**) and 278 (**h**). The C-2-methyl group can be assigned based on the required ion at *m*/*z* 101 and the absence of the respective ion **h**.

**Figure 6:**
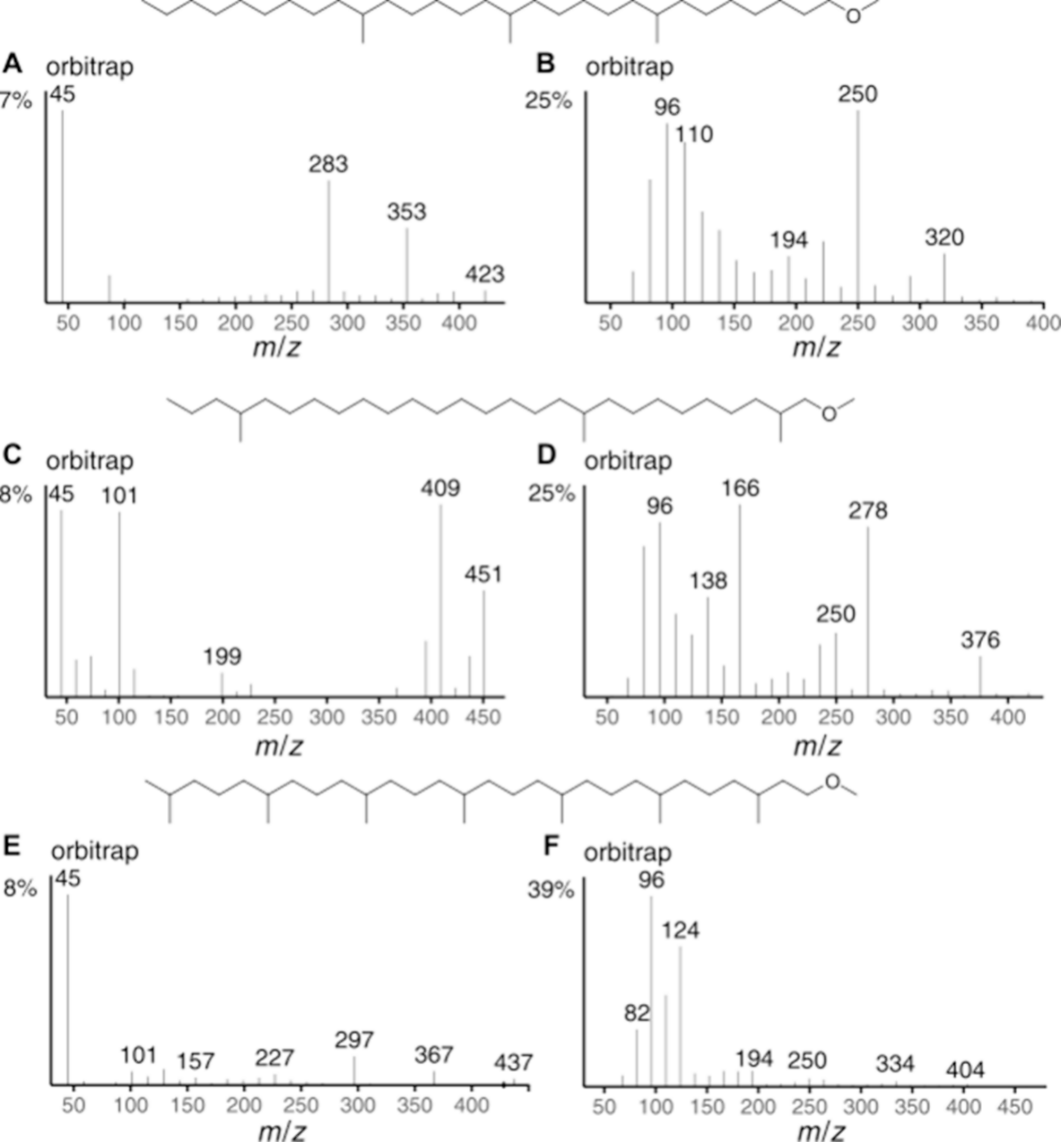
Orbitrap ISS45 (left) and ISS40 (right) of 1-methoxy-17,21,25-trimethylhexacosane (A, B), 1-methoxy-2,10,24-trimethylheptacosane (C, D), and 1-methoxy-3,7,11,15,19,23,27-heptamethyloctacosane (E, F).

Finally, highly branched 1-methoxy-3,7,11,15,19,23,27-heptamethyloctacosane (Figure 6E,F) was evaluated. The ions **a** with *m*/*z* 227, 297, 367, and 437 indicate the methyl groups between C-11 to C-23, while the isomethyl C-27 cannot be assigned. Ion **a** at *m*/*z* 157 indicates the C-7-methyl group, but its neighbors had similar intensities, preventing unequivocal allocation. The C-3-methyl cannot be directly assigned. The cyclic ion *m*/*z* 101 is formed as discussed, but the respective ion **h** is missing. Ions **d** and **h** are of lower intensity in the higher mass region, but support the assignment due to the characteristic occurrence of all required masses, *m*/*z* 124, 194, 264, 334, and 404 of **d**, and *m*/*z* 110, 180, 250, up to 320 in the **h** ion series.

The described analyses enable the assignment of methyl group positions in the aliphatic ethers found in spiders, as verified by the synthetic material. Some ambiguity remains between C-2 and C-4, but methyl branching at these positions can be clearly indicated by *m*/*z* = 101 and M-57 in the full mass spectra. Helpful is the prior determination of how many methyl groups can be expected, which can be deduced by calculation of gas chromatographic retention indices.^13–15^ A general procedure for performing the analyses is outlined in the Experimental section. In the following section, we describe the identification of naturally occurring methyl ethers in spider lipid extracts.

### Analysis of Natural Extracts of Spider Web Lipids

The epicuticular lipids of were identified previously as 1-methoxy-14,20-dimethylnonacosane and 1-methoxy-8,14,20-trimethylnonacosane.^13^ Their structures were deduced by GC/MS analysis of respective nitriles and methyl esters obtained by derivatization from the ethers.^11^ The identity of 1-methoxy-14,20-dimethylnonacosane was verified with a synthetic material. We then cross-checked the Orbitrap spectra of the ISS approach using these two ethers.

The ISS40 and ISS45 of 1-methoxy-8,14,20-trimethylnonacosane (Figure 7A,B) showed ions **a** at *m*/*z* 255 and 353, indicating methyl groups at C-14 and C-20. However, the ion at *m*/*z* 157 required for the C-8 methyl group was missing. In contrast, the respective ion **d** at *m*/*z* 124 showed a particularly high intensity, and together with ions **h** and **h**′**** at *m*/*z* 348 and 320, clearly indicated the C-8 branch. As reported, the gas chromatographic retention index was in agreement with that of a trimethyl ether.^13^ The ISS40 and ISS45 of the second natural ether, identified as 1-methoxy-14,20-dimethylnonacosane, matched those of the synthetic compound. However, an additional ion *m*/*z* 213 and enhanced intensities of ions *m*/*z* 180 and 278 indicated the presence of small amounts of 1-methoxy-12,20-dimethylnonacosane (Figure 7C,D). This minor constituent was not detected in the lipids by the derivatization procedure reported earlier.^13^

**Figure 7:**
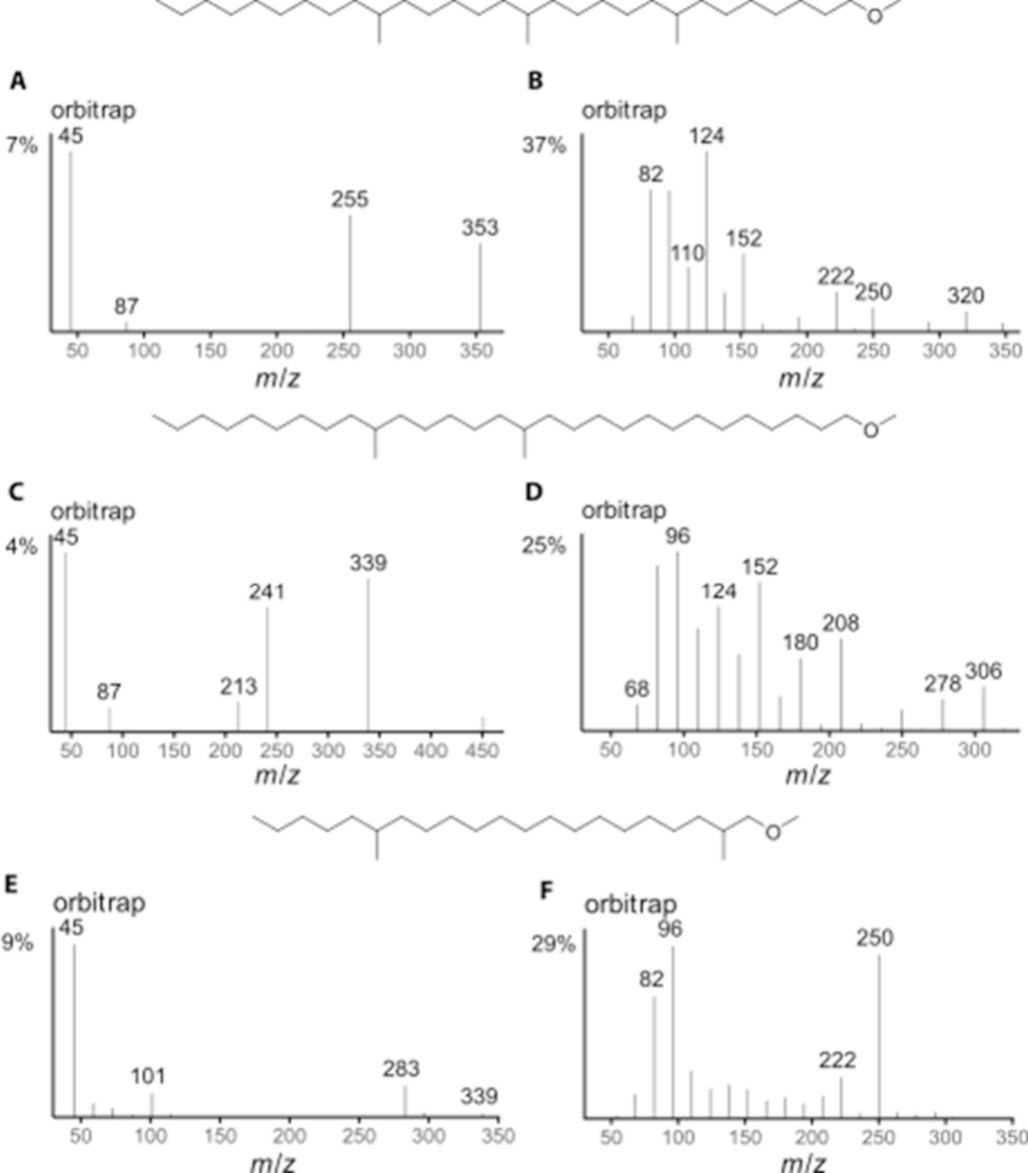
Orbitrap ISS45 (left) and ISS40 (right) of natural 1-methoxy-8,14,20-trimethylnonacosane (A, B) and 1-methoxy-14,20-dimethylnonacosane (C, D) of . (E, F) 1-methoxy-2,16-dimethylhenicosane of webs of male .

As a further application, a complex silk extract obtained from the spider (Linyphiidae) was investigated. Linyphid spiders are known to impregnate their webs with methyl ethers.^12,8^ The extracts of males contained a couple of long-chain ethers, also occurring in webs of females, and one shorter, male-specific ether (Supporting Information Figure S1). The long-chain ethers were identified along the lines discussed here, including information from experimental and calculated^15^ linear gas chromatographic retention indices. The identified compounds are listed in Table 1.

**Table 1:** Analytical Data of Methyl Ethers Occurring in Silk Extracts of Male ^a^

compound	rt	*I*	*I*_*calc*_	45	87	a	d	d**′**	h	h**′**	M-15
1-methoxy-2,16-dimethylhenicosane	2416	2419	2413	5.43	1.21	283 (1.73)	250 (26.77)	222 (6.84)	96 (28.40)		339 (0.16)
1-methoxy-26-methyloctacosane	29.19	3107	3105	8.05	2.20	409 (1.45)	376 (22.12)	348 (6.87)			
1-methoxy-26-methylnonacosane	29.73	3285	3284	6.45	2.32	409 (2.59)	376 (25.80)	348 (9.05)			437 (0.21)
1-methoxy-28-methyltriacontane	30.49	3308	3305	6.69	2.27	437 (1.26)	404 (22.18)	376 (6.27)			451 (0.62)
1-methoxy-28-methylhentriacontane	31.08	3387	3384	6.69	2.21	437 (2.25)	404 (22.76)	376 (8.97)			465 (0.13)
1-methoxy-2,28-dimethylhentriacontane	31.45	3427	3431	7.51	101 (1.32)	451 (0.62)	418 (21.81)	390 (4.88)	418 (21.81)	390 (4.88)	479 (0.05)

a *I*: experimental linear gas chromatographic retention index; *I*_*calc*_: calculated gas chromatographic retention index;^15^ 45, 87: *m*/*z*; a, d, d′, h, h′: fragments (see Scheme 1). Ion intensity in brackets.

The ISS40 and ISS45 of the male-specific compound are shown in Figure 7E and F. The ISS45 closely resembled that of synthetic 1-methoxy-4,16-dimethylhenicosane shown in Figure 5. However, the ISS40 was clearly different. The C-16 methyl can be confidently assigned using ions **a** (*m*/*z* 283) and **d**/**d**′**** (*m*/*z* 250, 222), as well as **h** (*m*/*z* 96) in both spectra. Another methyl group is located between C-2 and C-4 (*m*/*z* 101).

Ion **h** from C-4-methyl is present at *m*/*z* 278 in 1-methoxy-4,16-dimethylhenicosane, but it is lacking in the natural ether. A C-3-methyl seems unlikely because of the origin of the methyl ethers from the fatty acid biosynthetic pathway, placing all methyl groups uniformly on even-numbered carbons. In conclusion, the natural compound is 1-methoxy-2,16-dimethylhenicosane.

### Alcohols

The approach was further extended to alcohols, although we had only three synthetic compounds available (Figure 8). The ions **a** were of lower abundance compared to the methyl ethers and may easily be overlooked or not recorded at all if the concentration or spectrum quality is low. However, respective ions **d** and **h** were more intense. Analogs of *m*/*z* 87/101 of the methyl ether spectra are almost missing. 12-Methylheptacosan-1-ol showed *m*/*z* 199 (**a**) with only 1% intensity, indicating the C-12 methyl group (Figure 8A). However, this branching position was secured by intense ions *m*/*z* 180 (**d**) and 236 (**h**) (Figure 8B).

**Figure 8:**
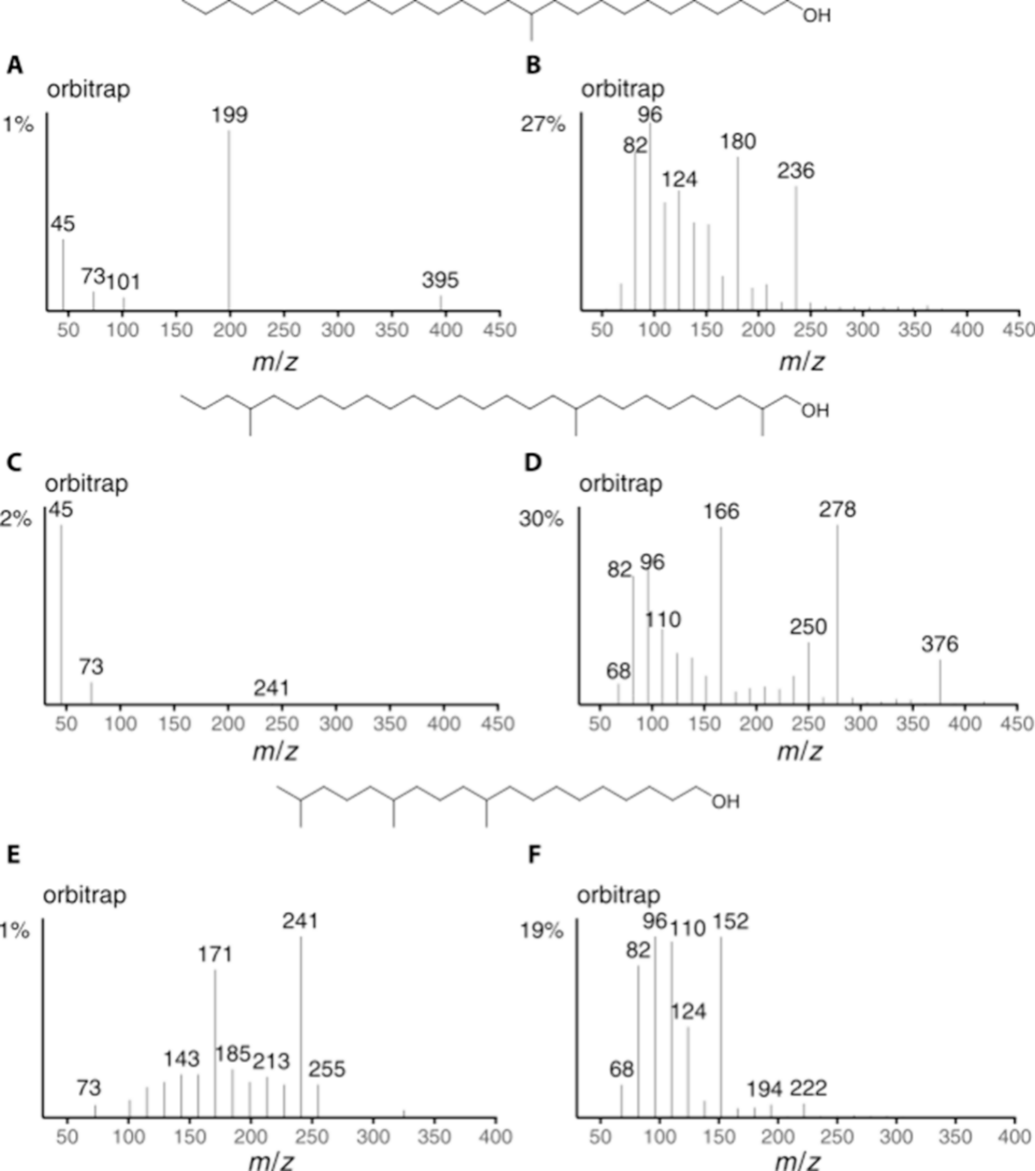
Orbitrap ISS45 (left) and ISS40 (right) of 12-methylheptacosan-1-ol (A, B), 2,10,24-trimethylheptacosan-1-ol (C, D), and 10,14,18-trimethylnonadecan-1-ol (E, F).

In the spectrum of 2,10,24-trimethylheptacosan-1-ol (Figure 8C,D), almost no **a** ion can be observed, although *m*/*z* 45 was of comparably high intensity, maybe by a rearrangement induced by the 2-methyl group. Nevertheless, the **d** ions *m*/*z* 166 and 376 and **h**/**h**′**** ions at *m*/*z* 278 and 250 revealed the positions of the methyl groups at C-10 and C-24. The 2-methyl group remained ambiguous and was not clearly deducible from the mass spectrum alone. However, the gas chromatographic retention index would reveal the presence of this methyl group. While an additional methylene group increases the retention index by 100, an additional 2-methyl group does this by only about 40.^14,15^

The spectrum of 10,14,18-trimethylnonadecan-1-ol (Figure 8E,F) showed the ions **a** at *m*/*z* 171 and 241, indicating the 10,14-dimethyl arrangement. Ions **d**/**d** **′** were clearly visible for the C-10-methyl group (*m*/*z* 152/124) but were only of very low abundance (*m*/*z* 222/194) for the C-14-methyl. The ion **h** at *m*/*z* 110 supported this localization, but the C-10 ion at *m*/*z* 180 was not significant. As discussed previously, the *iso*-methyl branch cannot be located with these spectra.

## Conclusions

The direct analyses of mass spectra of long-chain methyl-branched aliphatic methyl ethers obtained by GC/EI-Orbitrap MS allow the direct determination of methyl group positions along the aliphatic chain. This method seems reliable, especially when used in combination with gas chromatographic retention indices, which allow the assignment of the degree of methylation in the analyte. The method avoids derivatization, which is tedious, especially for natural extracts that are sometimes available only in small amounts, while the multiple chemical conversion steps necessary for an assignment are jeopardized by the small quantities and side products. The results are also more accurate because discrimination due to derivatization is avoided. Initial experiments suggest that this approach may also be applicable to other compound classes, as demonstrated for the respective alcohols; however, additional examples are needed.

## Supplementary Material





## Notes

Raw HR-MS data (XLSX)
Experimental, Excel and R macros, and unit-mass spectra (PDF)

## †

Chemistry Research Laboratory, University of Oxford, 12 Mansfield Road, Oxford, OX1 3TA, UK (A.M.)
