# Supplementary material for: Determination of Methyl Group Positions in Long-Chain Aliphatic Methyl Ethers and Alcohols by Gas Chromatography/Orbitrap Mass Spectrometry
Source: Anal Chem. 2025 Jul 29;97(31):17150–7. doi: 10.1021/acs.analchem.5c03083 (PMC12355476; doi:10.1021/acs.analchem.5c03083)
Supplement: Supplementary file 2 [file ac5c03083_si_002.pdf]

Supporting information for

Determination of Methyl Group Positions in Long-Chain Aliphatic Methyl Ethers and Alcohols by GC/Orbitrap Mass Spectrometry

Tatsuya Kiuchi,[a] Moritz Gerbaulet,[a] Anton Möllerke,[a,†] Tim Harig,[a] Axel Dinter,[b] Till Beuerle,[a] Stefan Schulz[a,*]

[a]Technische Universität Braunschweig, Institute of Organic Chemistry, Hagenring 30,
38106 Braunschweig, Germany.

[b]Nelkenweg 14, 61381 Friedrichsdorf, Germany

[†]Chemistry Research Laboratory, University of Oxford, 12 Mansfield Road, Oxford, OX1 3TA, UK.

Content

1 Generation of ion series mass spectra S2

2 GC/MS analysis of web extract of *Erigone atra* S5

3 Mass spectra S6

4 References S15

1 Generation of ion series mass spectra

Data were obtained using Xcalibur Qual Browser 2.2 SP1.48 (Thermo Scientific). The spectrum was selected and displayed as 'Spectrum List'. The elemental composition was calculated with the following isotope values: ^{14}N: 0, ^{16}O: 0-1, ^{12}C: 0-40, ^{13}C: 0-1, ^{1}H: 0-82. The exact mass values were then exported into the clipboard and copied into an Excel sheet. These raw data of all compounds discussed can be found in the accompanying Excel file, which contains all raw high-resolution mass spectrometry data. In Excel, the data were modified by two VBA macros. These macros were generated with the help of ChatGPT 4.0. The first macro '`DeleteFirstSixRows`' removed the first six lines in the active sheet, removed column B, transferred column A into nominal numbers, and removed all lines that do not contain a sum formula in the new column C. It is shown below:

```
Sub DeleteFirstSixRows()
    Dim ws As Worksheet
    Set ws = ActiveSheet ' Uses the active worksheet

    ' Delete the first six rows
    ws.Rows("1:6").Delete Shift:=xlUp

    ' Delete column B
    ws.Columns(2).Delete

    ' Declare variables
    Dim lastRow As Long
    Dim i As Long
    Dim cellValue As String
    Dim posDot As Integer

    ' Determine the last used row in column C
    lastRow = ws.Cells(ws.Rows.Count, 3).End(xlUp).Row

    ' Loop through the rows from bottom to top
    For i = lastRow To 1 Step -1
        ' Check if the cell in column C is empty
        If IsEmpty(ws.Cells(i, 3).Value) Then
            ' Delete the row if column C is empty
            ws.Rows(i).Delete
        End If
    Next i

    ' Determine the last used row in column A (again, after possible
deletions)
    lastRow = ws.Cells(ws.Rows.Count, 1).End(xlUp).Row

    ' Loop through the rows from top to bottom
    For i = 1 To lastRow
        cellValue = ws.Cells(i, 1).Value
        posDot = InStr(cellValue, ".")

        ' Check if a dot is present in the string
        If posDot > 0 Then
            ' Remove everything after the dot, including the dot itself
            ws.Cells(i, 1).Value = Left(cellValue, posDot - 1)
        End If
    Next i
End Sub
```

The second macro, 'DeleteInappropriateRows', removed all lines that were not consistent with a wanted ion series. The macro requires input for the selected ion series. Therefore, a value must first be added in field A1 of the Excel sheet, e.g., 45 for ISS45 ($C_nH_{2n+1}O$). Then the second macro can be applied. Sometimes it is helpful to introduce an additional line above the data before doing this. An example of the output is hsown in Tabel S1.

```
Sub DeleteInappropriateRows()
    Dim ws As Worksheet
    Dim lastRow As Long
    Dim i As Long
    Dim cellValue As Variant
    Dim baseValue As Long

    ' Uses the active worksheet
    Set ws = ActiveSheet

    ' Read the value in cell A1
    baseValue = ws.Cells(1, 1).Value

    ' Determine the last row in column A
    lastRow = ws.Cells(ws.Rows.Count, 1).End(xlUp).Row

    ' Check the rows from bottom to top
    For i = lastRow To 2 Step -1
        cellValue = ws.Cells(i, 1).Value

        ' Check if the cell value equals 45 + N*14
        If Not IsNumeric(cellValue) Or (cellValue - baseValue) Mod 14 <> 0
Or (cellValue - baseValue) < 0 Then
            ' If not, delete the row
            ws.Rows(i).Delete
        End If
    Next i
End Sub
```

The data can now be checked using Excel's diagram options. In some cases, it can be advantageous to manually remove some remaining values that do not match the ion series, although the abundance of these ions is usually very small.

However, we were not fully satisfied with the Excel diagram possibilities and opted instead to use an R program for plotting mass spectra that we developed. This program was improved and modified with the help of ChatGPT 4.0. It requires the package 'ggplot2'.[S1] The data in the Excel sheet were then loaded into R.

```
library(ggplot2)

ms_plot <- function(df, displayed_labels = 5, name = "test", RI = "orbitrap",
text_size = 12, xmax_value = NULL) {
  # Assign column names
  ColNames <- c("m/z", "Intensity")
  colnames(df) <- ColNames

  # Convert columns to numeric types
  df$`m/z` <- as.numeric(df$`m/z`)
  df$Intensity <- as.numeric(df$Intensity)

  # Function for "nice" rounding up
  roundUpNice <- function(x, nice = c(seq(1, 6, by = 0.1))) {
    if (length(x) != 1) stop("'x' must be of length 1")
    10^floor(log10(x)) * nice[[which(x <= 10^floor(log10(x)) * nice)[1]]]
```

```
  }

  # Set the xmax value based on the new parameter or the maximum value in the data
  if (is.null(xmax_value)) {
    xmax_value <- roundUpNice(max(df$`m/z`, na.rm = TRUE)) + 10
  }

  # Labeling for the highest MS peaks
  highest_I <- order(df$Intensity, decreasing = TRUE)[1:displayed_labels]
  xlabels <- rep("", nrow(df))
  xlabels[highest_I] <- round(df$`m/z`[highest_I])

  # Determine maximum intensity and adjust Y-axis label
  max_intensity <- ceiling(max(df$Intensity, na.rm = TRUE))
  y_label <- paste0(max_intensity, "%")

  # Axis text
  xtext <- expression(paste(italic("m"), "/", italic("z")))

  # Plot data
  ms <- ggplot(df, aes(x = `m/z`, y = Intensity)) +
    labs(subtitle = RI) +
    geom_col(width = 1) +
    scale_x_continuous(
      name = xtext,
      expand = c(0, 0),
      limits = c(30, xmax_value + 1),
      breaks = seq(0, xmax_value, by = 50)
    ) +
    scale_y_continuous(
      name = y_label,
      expand = c(0, 0),
      limits = c(0, 1.1 * max(df$Intensity, na.rm = TRUE)),
      breaks = NULL
    ) +
    theme(
      axis.line.x = element_line(colour = "black", size = 0.5),
      axis.line.y = element_line(colour = "black", size = 0.5),
      axis.title.x = element_text(size = text_size, vjust = -1.5),
      axis.title.y = element_text(size = text_size, angle = 0),
      plot.subtitle = element_text(size = text_size, vjust = 0.5),
      axis.text.y = element_blank(),
      axis.ticks.x = element_line(colour = "black", size = 0.5),
      panel.grid.major = element_blank(),
      panel.grid.minor = element_blank(),
      panel.border = element_blank(),
      panel.background = element_blank()
    ) +
    geom_text(aes(label = xlabels), vjust = -0.5, color = "black", position =
position_dodge(width = 0.5), size = text_size * 0.3)

  print(ms)

  # Save plot
  format <- ".pdf"
  height <- 2
  suppressWarnings(ggsave(file = paste0(name, format), plot = ms, width = 1.6180339887
* height, height = height))
}

# Create example data (without column headers)
df <- data.frame(
  seq(50, 500, 10),
  runif(46, min = 0, max = 100)
)

# Apply the function with a custom xmax_value
ms_plot(df, displayed_labels = 15, name = "Sample_MS", RI = "Label", text_size = 14,
xmax_value = 600)
```

The program can take up some variables. Number of peaks containing numbers: `displayed_labels`; name of output file: `name`; character field in the upper left corner: `RI`, letter size, standard 12 pt: `text_size`, length of x-axis: `xmax_value`. The output is directly stored in a PDF file.

Table S1. Example Excel Data sheet after running the described VBA macros.

m/z	Intensity	
40	10.32	C3 H5
54	0.15	C4 H6
68	2.27	C5 H8
82	9.3	C6 H10
96	27.2	C7 H12

2 GC/MS analysis of web extract of *Erigone atra*

Five webs of three-week-old mated male and female *Erigone atra* (Linyphiidae) spiders were pooled and analyzed with GC/MS. The results are shown in Figure S1. The analysis showed that the relatively short 1-methoxy-2,16-dimethylhenicosane is male-specific, while the other methyl ethers occur in both sexes.

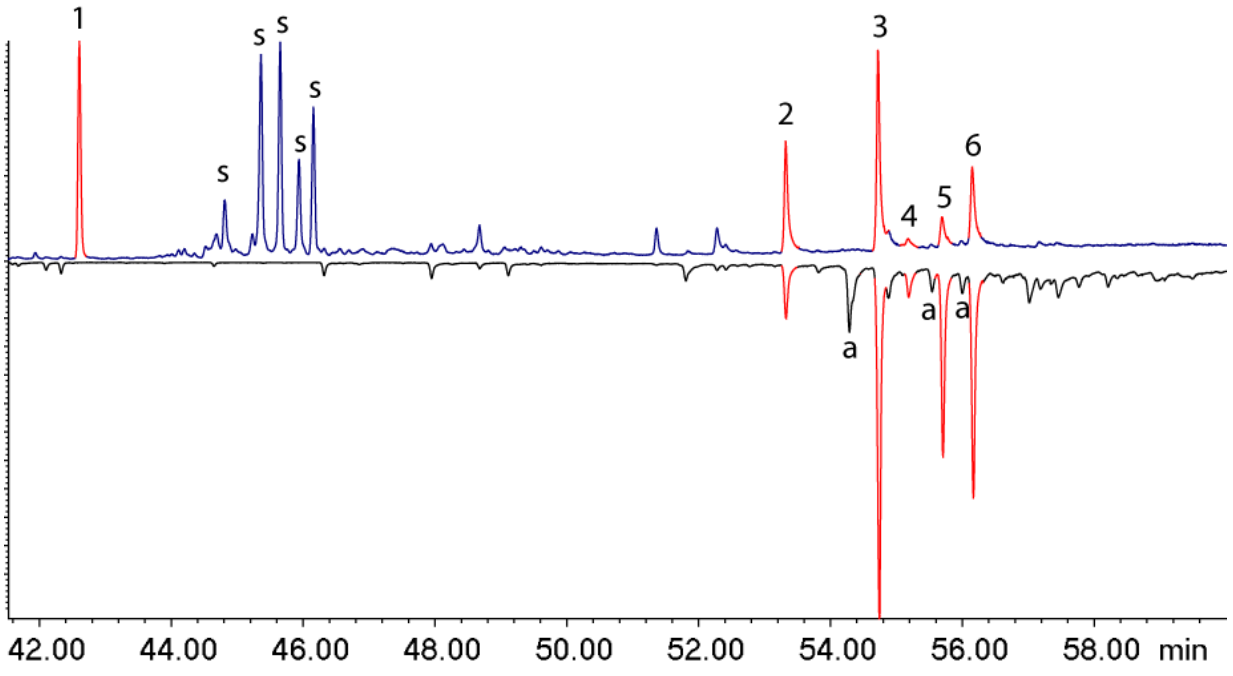

Figure S1. TICs of web extracts of *Erigone atra*. The upper chromatogram is from males, the lower one from females. Methyl ethers are indicated in red. 1: 1-methoxy-26-methyloctacosane; 2: Other peaks are either impurities, steroids, or linear hydrocarbons. S: steroids; a: long-chain unsaturated hydrocarbons; 2: 1-methoxy-26-methylnonacosane; 3: 1-methoxy-28-methyltriacontane; 4: 1-methoxy-28-methylhentriacontane; 5: 1-methoxy-2,28-dimethylhentriacontane

3 Mass spectra

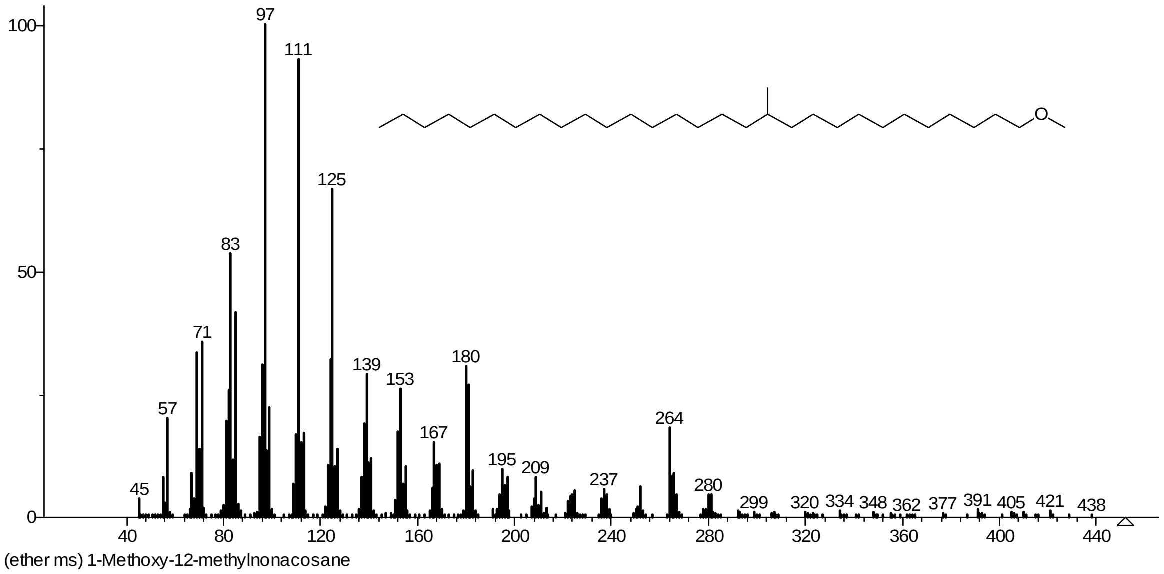

Figure S2. Orbitrap mass spectrum (nominal mass) of 1-methoxy-12-methylnonacosane.

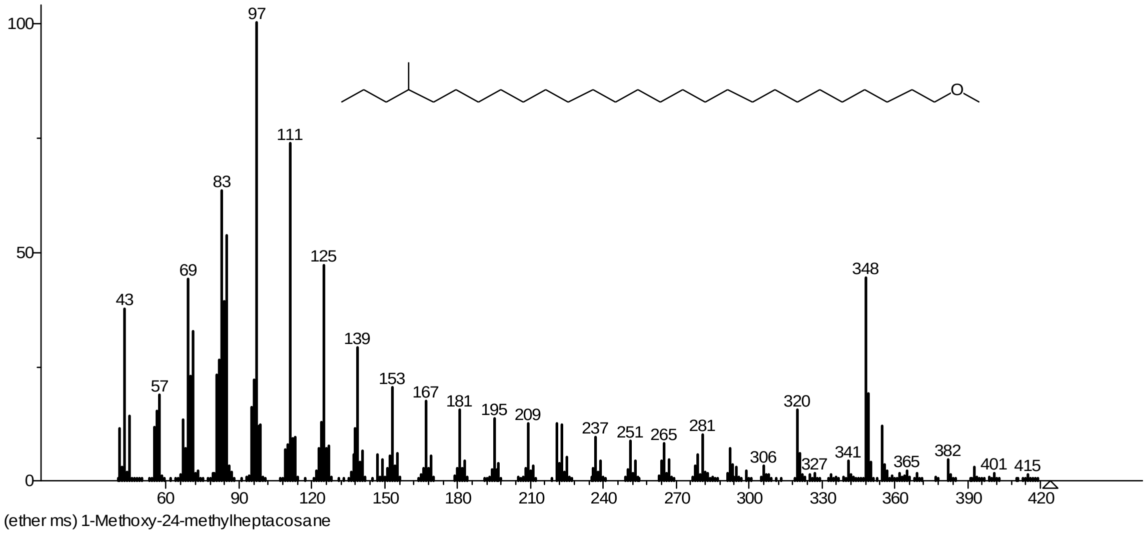

Figure S3. Orbitrap mass spectrum (nominal mass) of 1-methoxy-24-methylheptacosane.

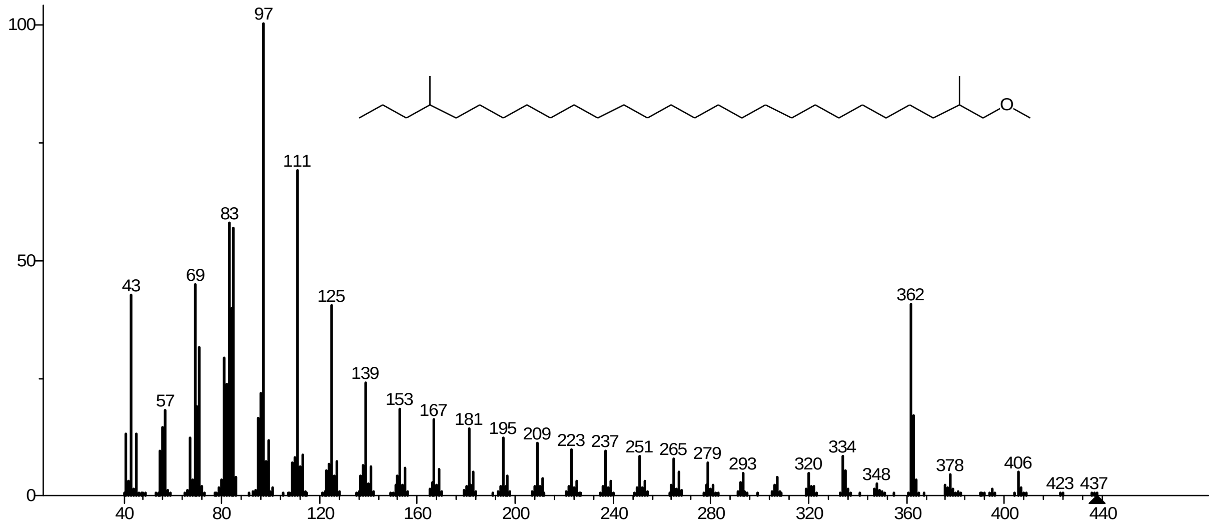

(ether ms) 1-Methoxy-2,24-dimethylheptacosane

Figure S4. Orbitrap mass spectrum (nominal mass) of 1-methoxy-2,24-dimethylheptacosane.

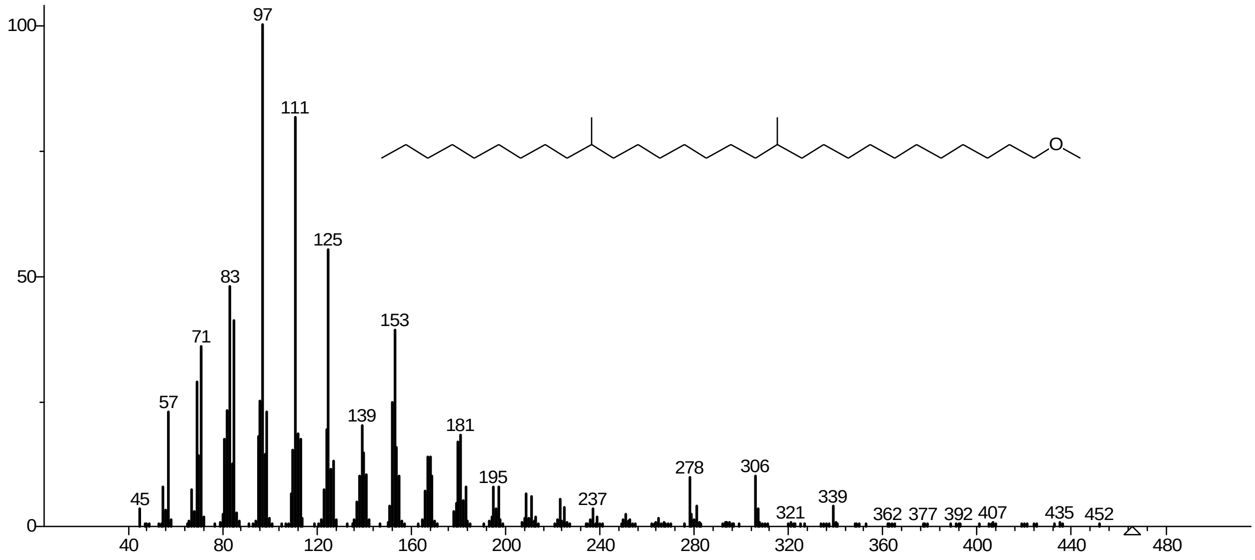

(Spec. Edit) 1-Methoxy-12,20-dimethylnonacosane

Figure S5. Orbitrap mass spectrum (nominal mass) of 1-methoxy-12,20-dimethylnonacosane.

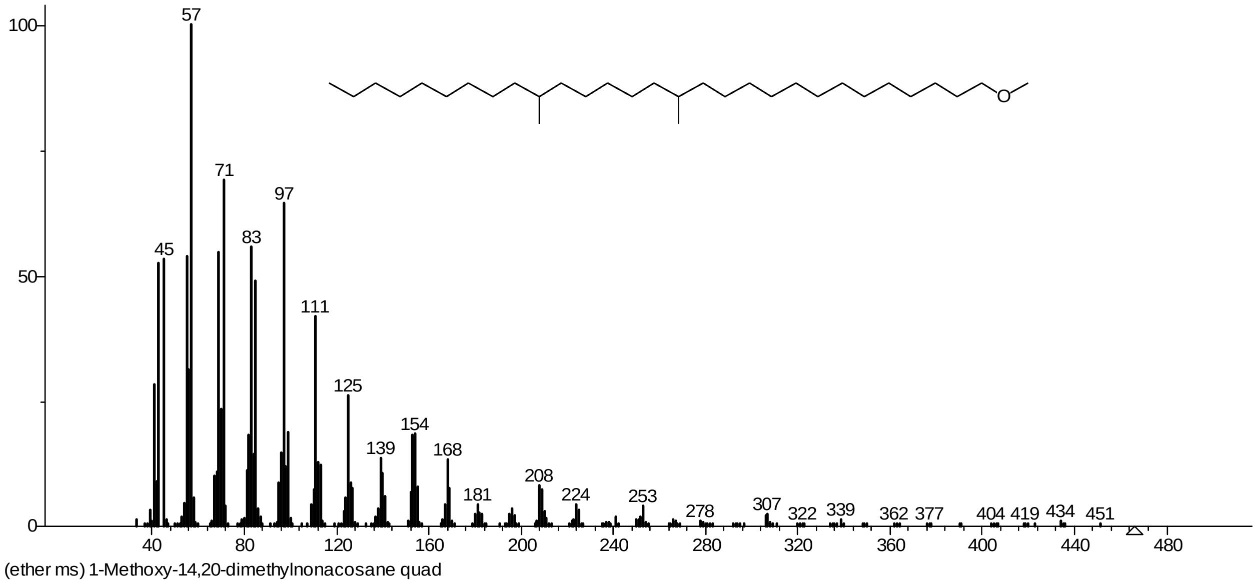

Figure S6. Orbitrap mass spectrum (nominal mass) of 1-methoxy-14,20-dimethylnonacosane.

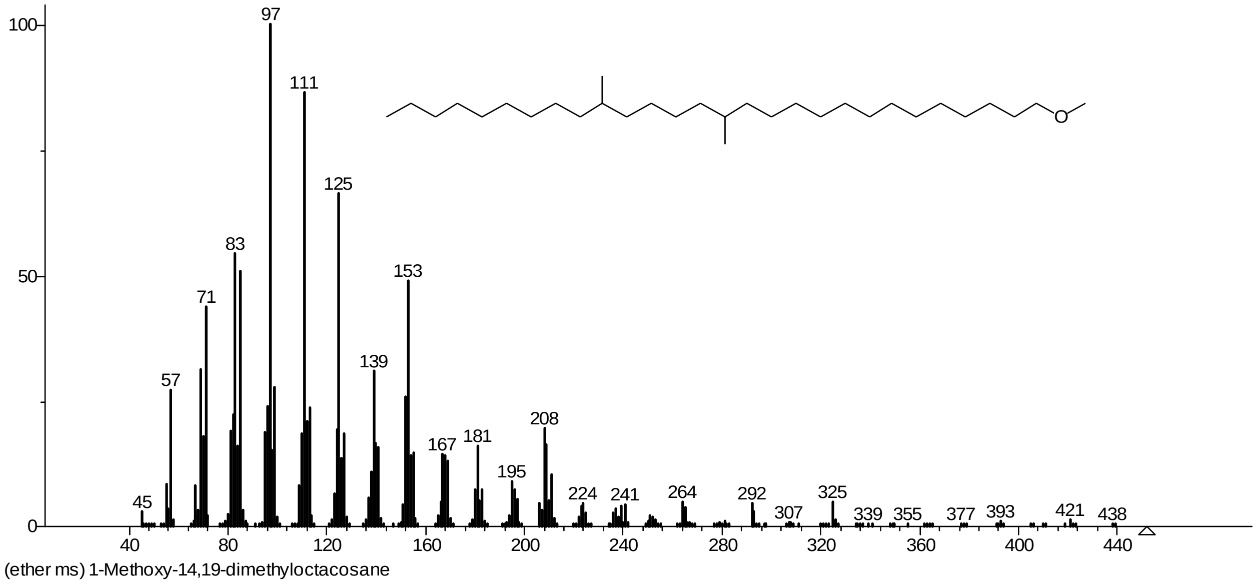

Figure S7. Orbitrap mass spectrum (nominal mass) of 1-methoxy-14,19-dimethyloctacosane.

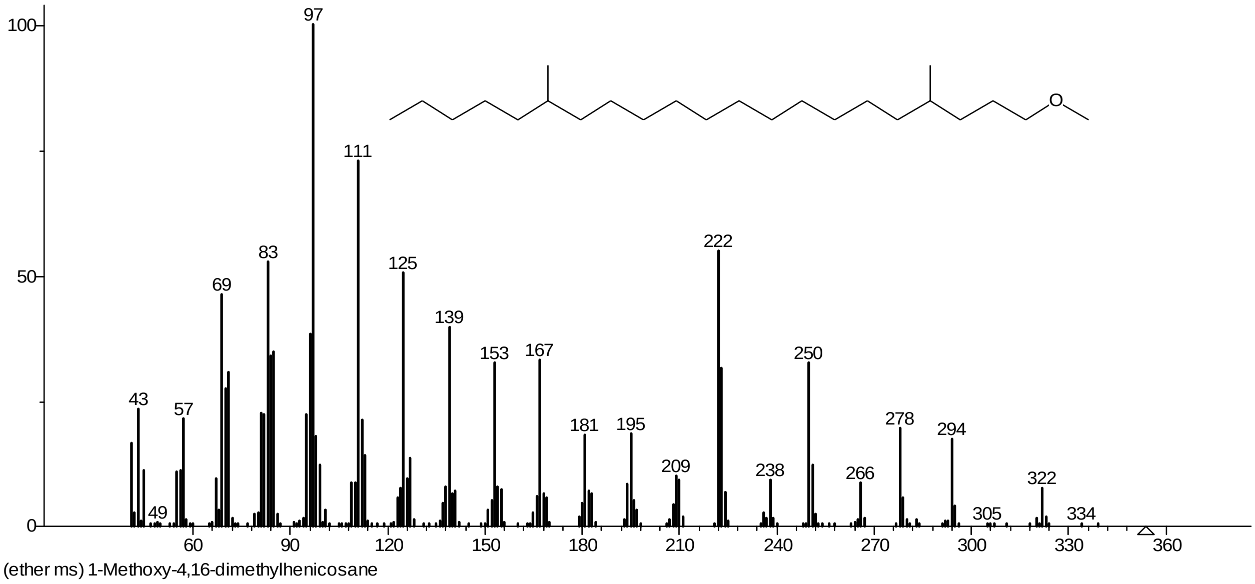

Figure S8. Orbitrap mass spectrum (nominal mass) of 1-methoxy-4,16-dimethylhenicosane.

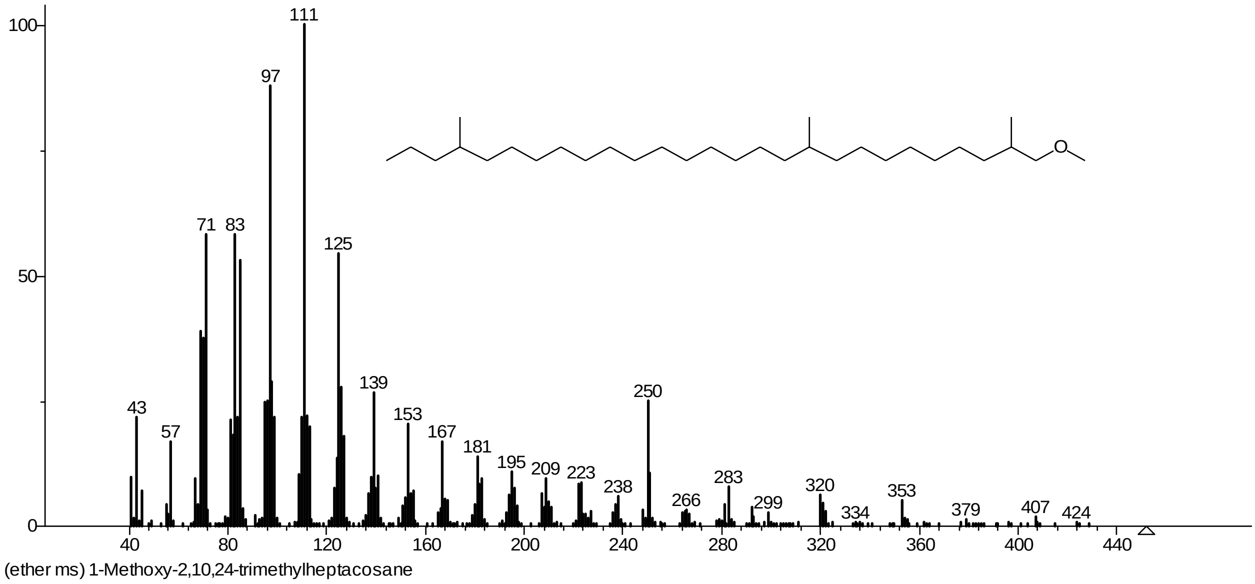

Figure S9. Orbitrap mass spectrum (nominal mass) of 1-methoxy-2,10,24-trimethylheptacosane.

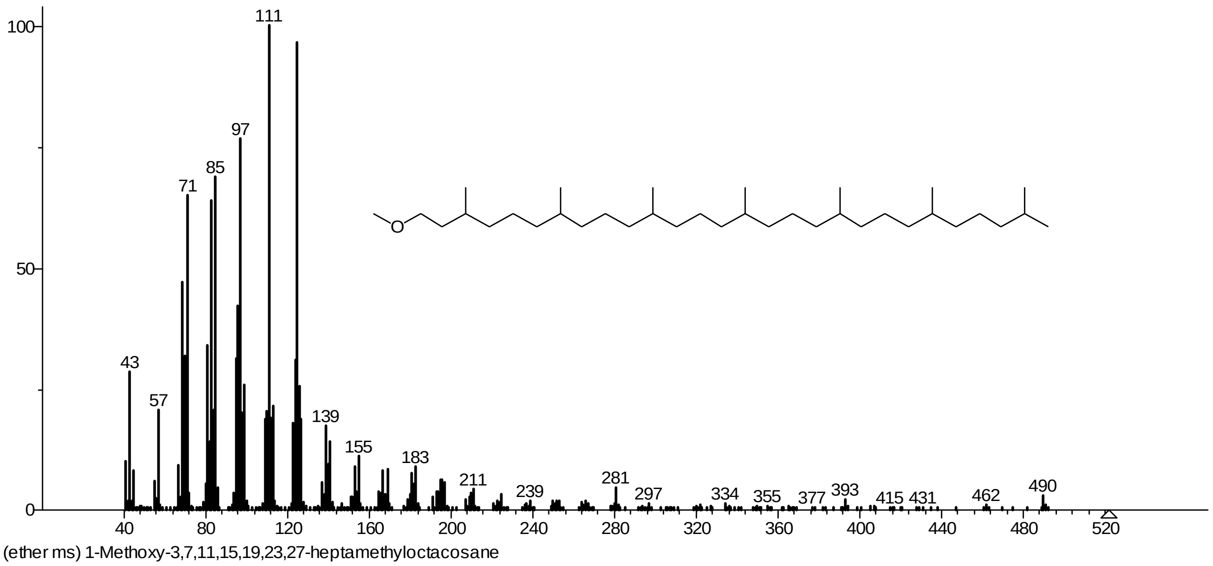

Figure S10. Orbitrap mass spectrum (nominal mass) of 1-methoxy-3,7,11,15,19,23,27-heptamethyloctacosane.

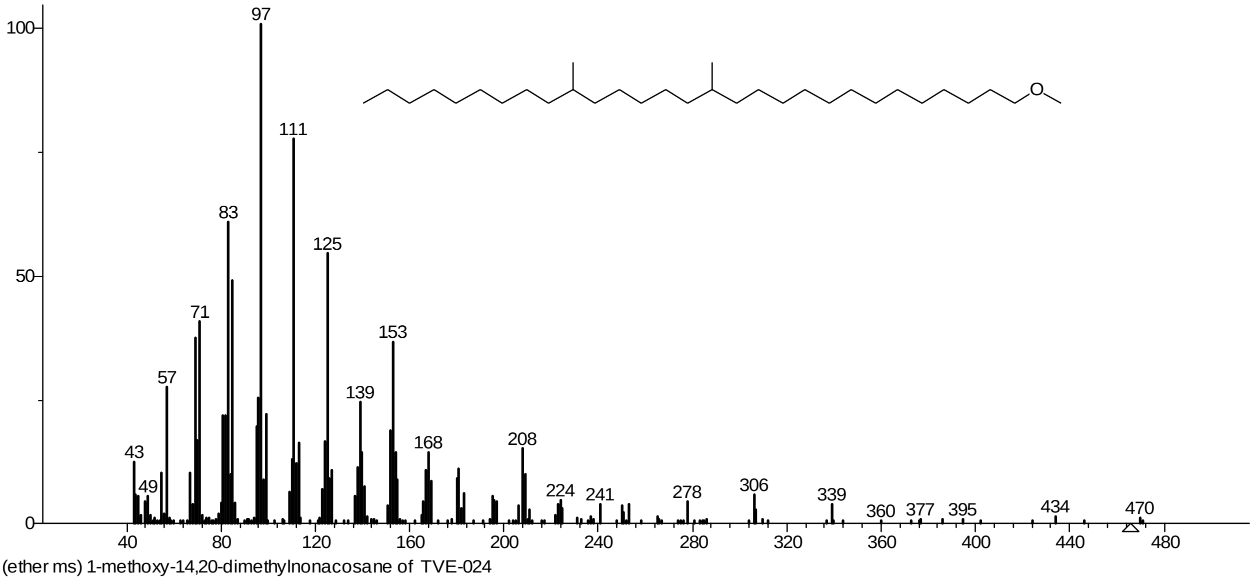

Figure S11. Orbitrap mass spectrum (nominal mass) of 1-methoxy-14,20-dimethylnonacosane from the epicuticular lipids of *Tetragnatha versicolor*.

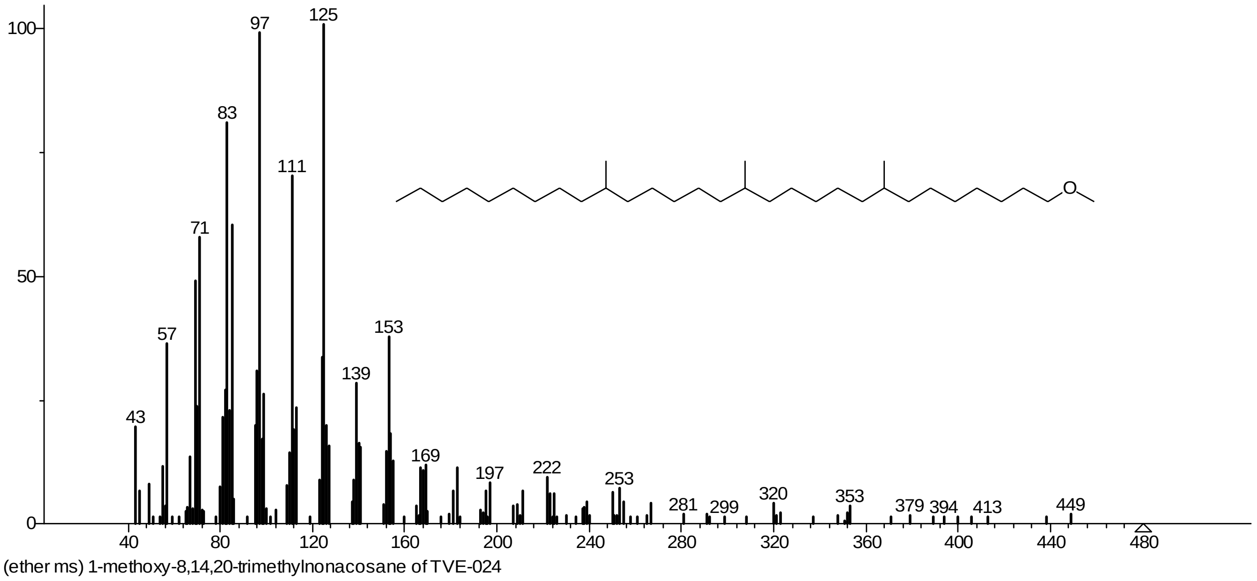

Figure S12. Orbitrap mass spectrum (nominal mass) of 1-methoxy-8,14,20-trimethylnonacosane from the epicuticular lipids of *Tetragnatha versicolor*.

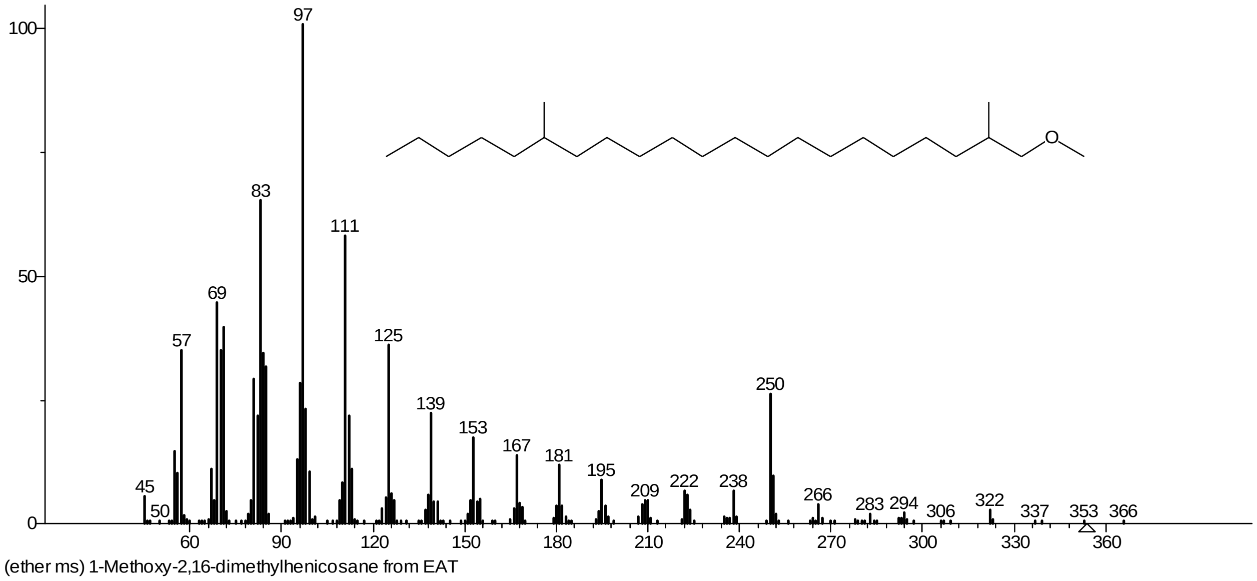

Figure S13. Orbitrap mass spectrum (nominal mass) of 1-methoxy-2,16-dimethylhenicosane from the webs of male *Erigone atra*.

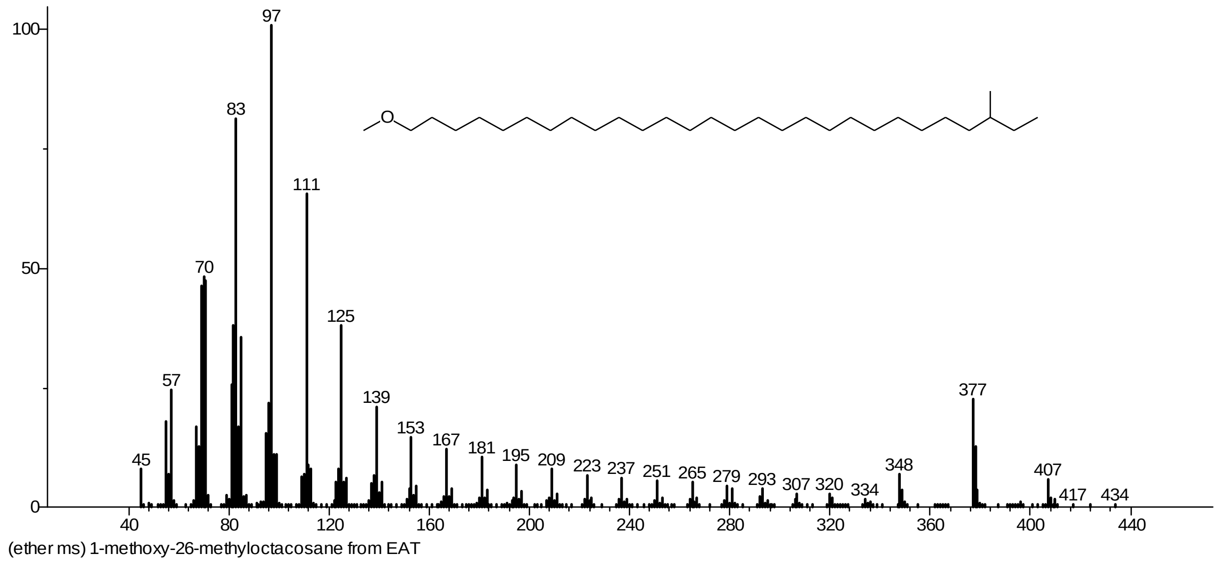

Figure S14. Orbitrap mass spectrum (nominal mass) of 1-methoxy-26-methyloctacosane from the webs of male *Erigone atra*.

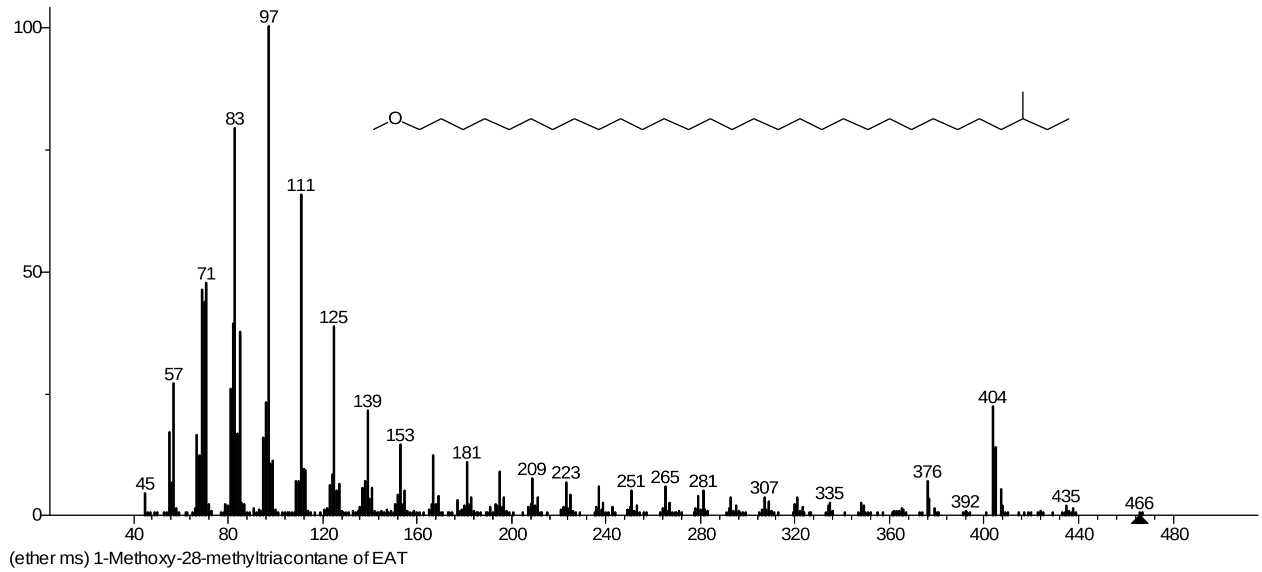

Figure S15. Orbitrap mass spectrum (nominal mass) of 1-methoxy-28-methyltriacontane from the webs of male *Erigone atra*.

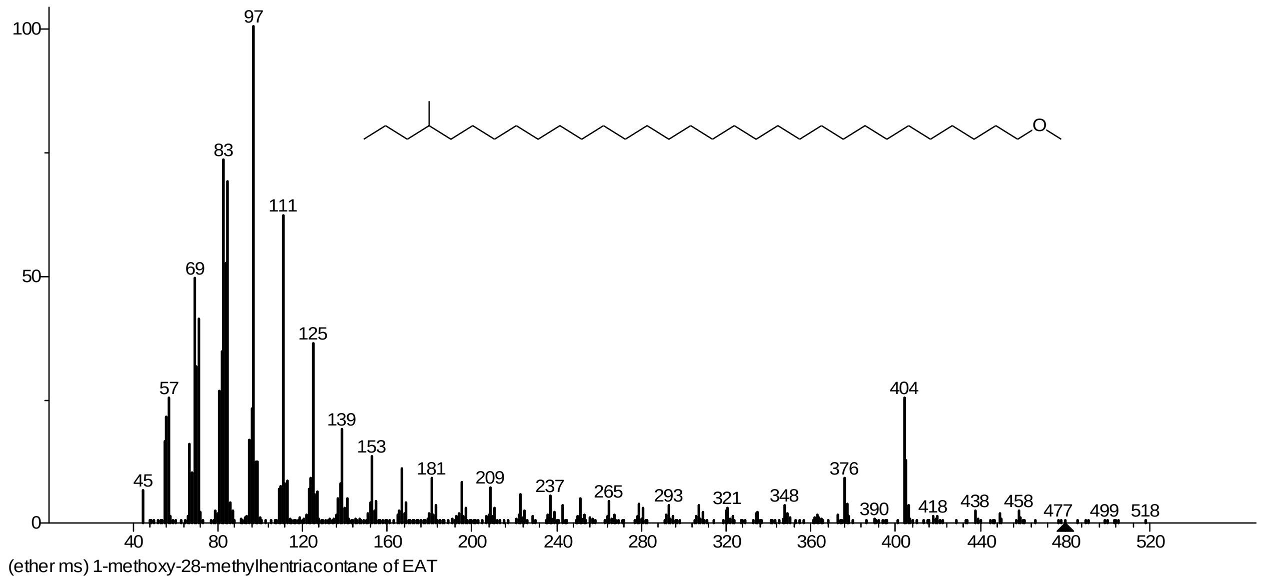

Figure S16. Orbitrap mass spectrum (nominal mass) of 1-methoxy-28-methyltriacontane from the webs of male *Erigone atra*.

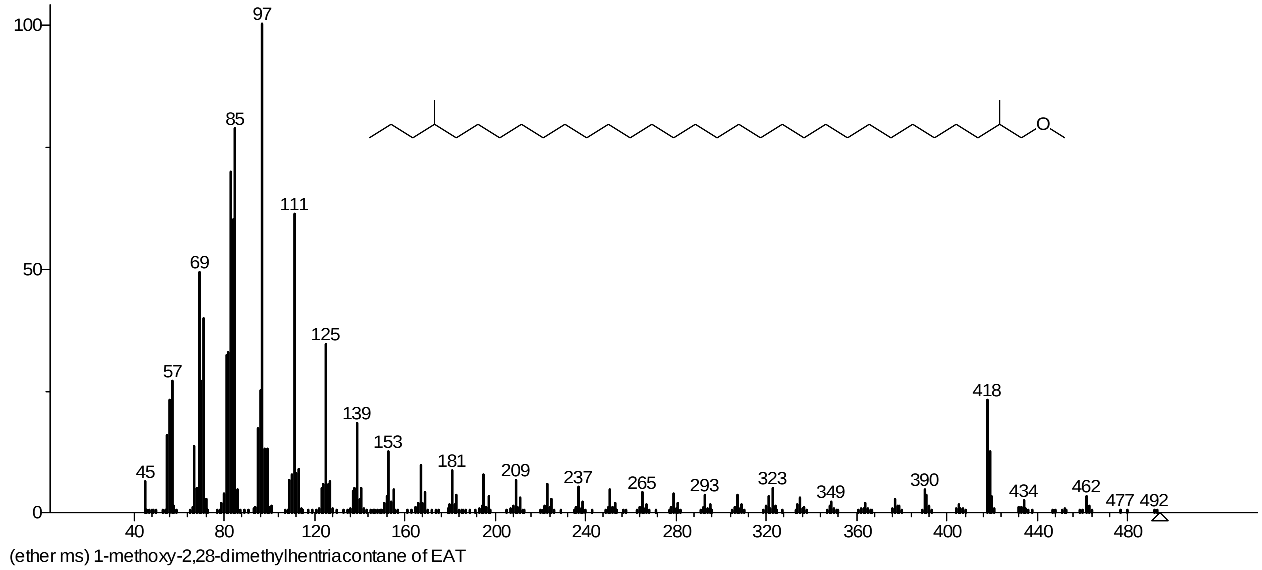

Figure S17. Orbitrap mass spectrum (nominal mass) of 1-methoxy-2,28-dimethylhentriacontane from the webs of male *Erigone atra*.

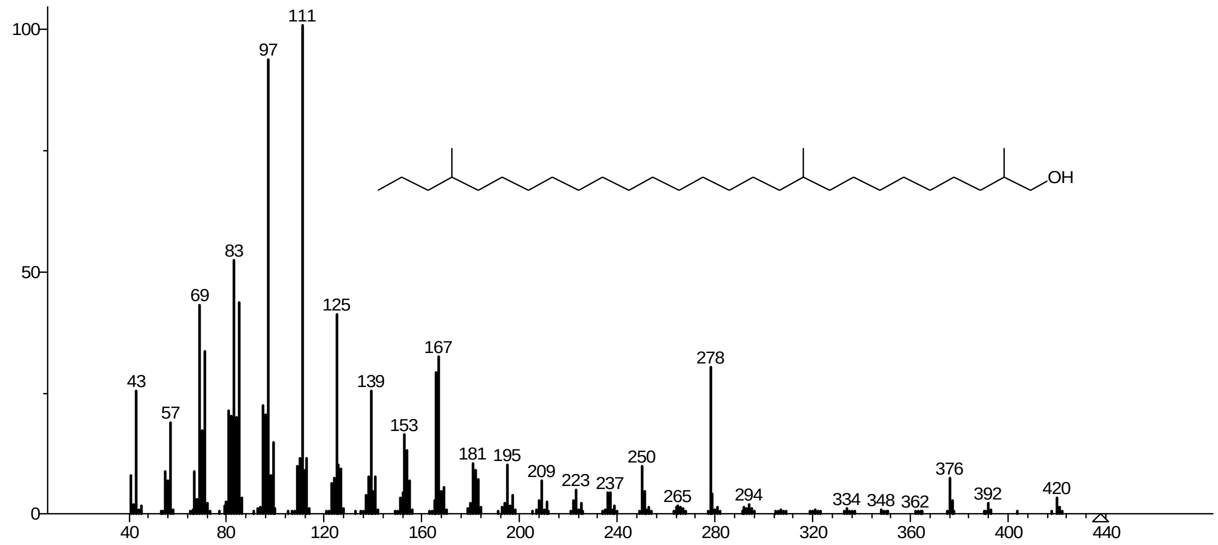

(ether ms) 2,10,24-Trimethylheptacosan-1-ol

Figure S18. Orbitrap mass spectrum (nominal mass) of 2,10,24-trimethylheptacosan-1-ol.

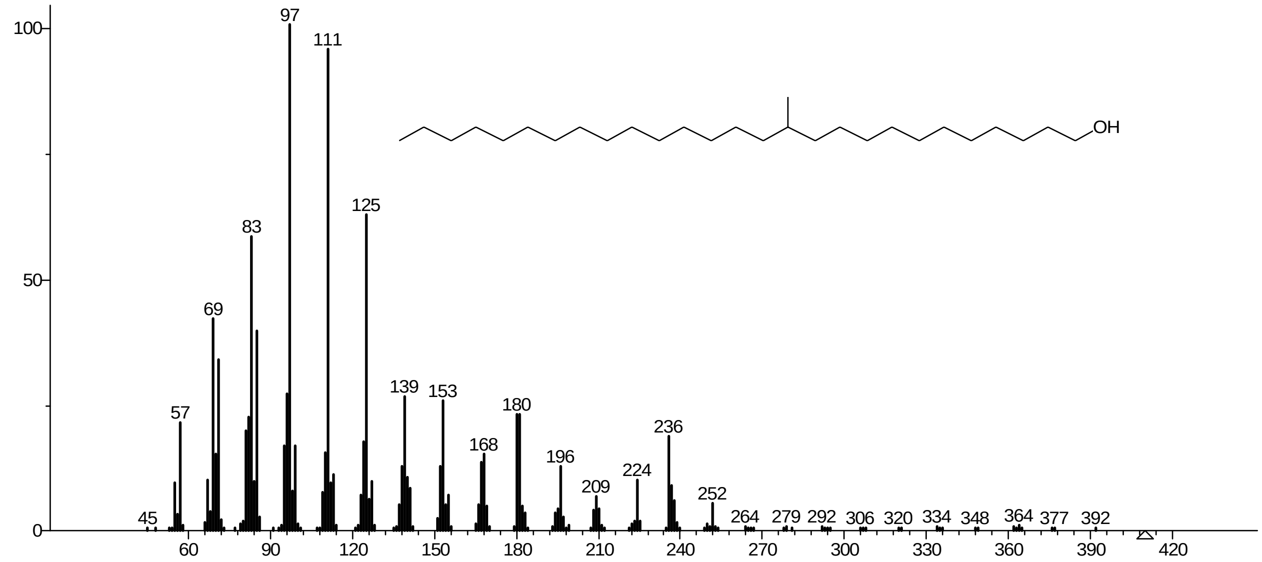

(ether ms) 12-Methylheptacosan-1-ol

Figure S19. Orbitrap mass spectrum (nominal mass) of 12-methylheptacosan-1-ol.

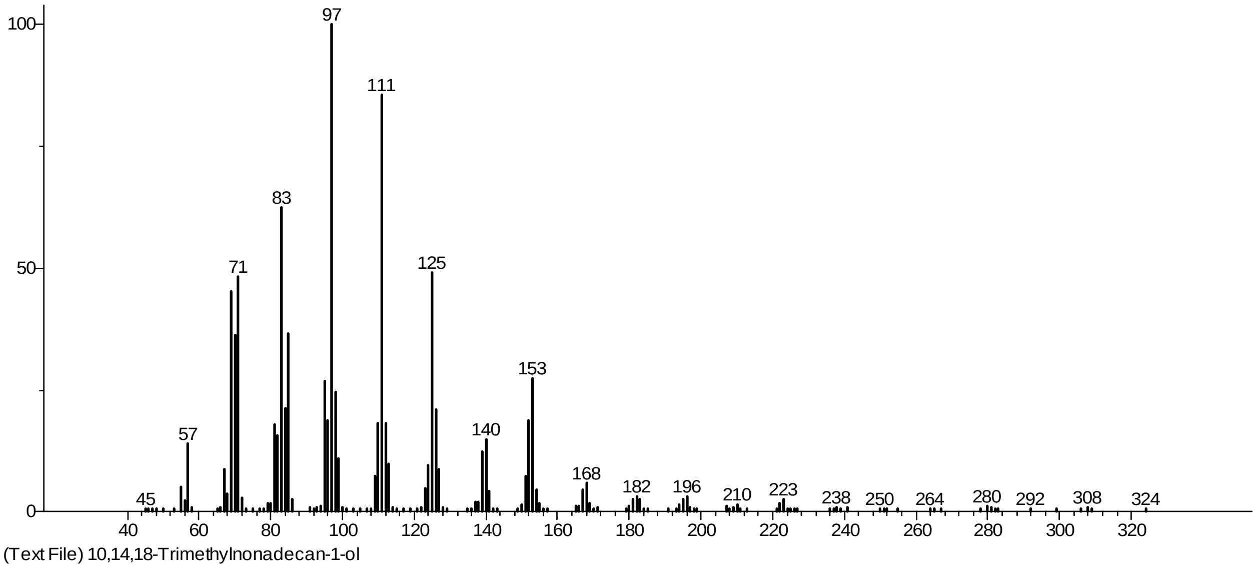

Figure S20. Orbitrap mass spectrum (nominal mass) of 10,14,18-nonadecan-1-ol.

4 References

(S1) Wickham, H. *ggplot2: Elegant graphics for data analysis*, Second edition; Use R!; Springer International Publishing: Cham, 2016.
